# Comprehensive pan‑cancer analysis of potassium voltage-gated channel Q4 (KCNQ4) gene across multiple human malignant tumors

**DOI:** 10.1038/s41598-023-45074-7

**Published:** 2023-10-30

**Authors:** Qing Zhao, Meizeng Li, Yunxiang Zhang

**Affiliations:** 1 Pathology Department, First Affiliated Hospital of Weifang Medical University (Weifang People’s Hospital), Weifang, China; 2 Department of Basic Medicine, Weifang Medical University, Weifang, China

**Keywords:** Cancer, Computational biology and bioinformatics, Immunology

## Abstract

A large number of studies indicate that Potassium Voltage-Gated Channel Q4 (KCNQ4) gene is the cause of non-syndromic hearing loss, but there are few studies investigating the role of KCNQ4 in cancers and scarcity of comprehensive analysis of its involvement in the diagnosis, methylation, mutation, prognosis of various cancer types. Therefore, the aim of this study is to examine the anticancerous and immune effects of KCNQ4 in various cancers and its potential value in breast cancer. In this study, we explored the potential role of KCNQ4 in cancers using public databases and the R software for bioinformatics analysis. The results showed that the low expression of KCNQ4 across specific cancer types was positively associated with low mutation frequency and methylation, and the improved survival. Eight small molecule compounds were identified that could potentially target KCNQ4. In addition, immunohistochemistry confirmed that the KCNQ4 expression was low in breast cancer. In *vitro* experiments confirmed that overexpression of KCNQ4 inhibited cell migration and invasion and promoted apoptosis. In summary, our comprehensive pan-cancer analysis highlights the potential of KCNQ4 as a cancer marker, and can be used as an auxiliary prognostic indicator and an indicator for immunotherapy in certain tumor types.

## Introduction

Malignant tumors pose a significant threat to human health and have become the disease with the leading cause of mortality in the world^1^. Although chemotherapy and immunotherapy prolong patients' overall survival and improve their quality of life, tumors often recur due to the ineffective long-term antitumor responses^2, 3^. Therefore, it is imperative to investigate the pan-cancer analysis of genes in malignant tumors and their role in specific cancer types.

Potassium voltage-gated channel subfamily Q Member 4(KCNQ4) is a homologous tetramer protein located in the LP34 region of human chromosome 1. It consists of six transmembrane domains, one pore region, and two intracellular domains (Supplementary Fig. 1). KCNQ4 encodes a protein that forms a potassium channel that was originally thought to play a crucial role in regulating neuronal excitability, particularly in the sensory hair cells of the cochlea^4–7^. In recent years, several studies reported the role of KCNQ4 in several malignant tumors. For instance, previously described in the study of Zhang et al.^8^, KCNQ4 can be up-regulated by BC069792 to inhibit the growth in breast cancer(BRCA). Sevilla-Montero et al.^9^ recently surveyed the expression of KCNQ4 in blood vessels was significantly lower than non-smoking patients and smoking patients in chronic obstructive pulmonary disease (COPD), which is an “accomplice ’’ of lung adenocarcinoma (LUAD) . Inagaki et al.^10^ recently surveyed in rat rectal colon (RC), KCNQ4 can activate intestinal secretion and play a role in defense mechanism. Santos et al.^11^ recently surveyed that the expression of KCNQ4 was lower than normal tissue and KCNQ4 of overexpression had a short time for biochemical recurrence in prostate adenocarcinoma (PRAD). However, a pan-cancer analysis of KCNQ4 based on clinical big data has not been reported. Public databases such as the Cancer Genome Atlas (TCGA) provide functional genomics information for different cancers (Table 1), enabling pan-cancer analysis on any gene of interest.

**Table 1:** Cancer abbreviations and the corresponding full name of abbreviations.

ACC	Adrenocortical carcinoma
BLCA	Bladder urothelial carcinoma
BRCA	Breast invasive carcinoma
CESC	Cervical squamous cell carcinoma and endocervical adenocarcinoma
CHOL	Cholangiocarcinoma
COAD	Colon adenocarcinoma
COADREAD	Colon adenocarcinoma/rectum adenocarcinoma esophageal carcinoma
DLBC	Lymphoid neoplasm diffuse large B-cell lymphoma
ESCA	Esophageal carcinoma
GBM	Glioblastoma multiforme
GBMLGG	Glioma
HNSC	Head and neck squamous cell carcinoma
KICH	Kidney chromophobe
KIPAN	Pan-kidney cohort (KICH + KIRC + KIRP)
KIRC	Kidney renal clear cell carcinoma
KIRP	Kidney renal papillary cell carcinoma
LAML	Acute myeloid leukemia
LGG	Brain lower grade glioma
LIHC	Liver hepatocellular carcinoma
LUAD	Lung adenocarcinoma
LUSC	Lung squamous cell carcinoma
MESO	Mesothelioma
OV	Ovarian serous cystadenocarcinoma
PAAD	Pancreatic adenocarcinoma
PCPG	Pheochromocytoma and paraganglioma
PRAD	Prostate adenocarcinoma
READ	Rectum adenocarcinoma
SARC	Sarcoma
STAD	Stomach adenocarcinoma
SKCM	Skin cutaneous melanoma
STES	Stomach and esophageal carcinoma
TGCT	Testicular germ cell tumors
THCA	Thyroid carcinoma

In this study multiple databases were used to explore the expression level, methylation, gene mutation, prognosis, immune infiltration, tumor stemness, related pathways and potential drugs of malignant tumors, Our main objective was to uncover the potential function of KCNQ4 using bioinformatics in various tumors and verify its potential influence on the development of breast cancer. Our results revealed the low expression of KCNQ4 in breast cancer. Moreover, overexpression of KCNQ4 inhibited the migration, proliferation and invasion, while promoting the apoptosis of breast cancer cells. These findings provide novel insights into KCNQ4 as a potential therapeutic target for cancer treatment.

## Materials and methods

### Cell lines, cell culture and transfection

The breast cancer cell lines MDA-MB-231 and MDA-MB-468 were purchased from ATCC cell bank and cultured in RPMI-1640 medium. Heat-inactivated fetal bovine serum (10%) was added to the above culture medium, and cultured in a 5% CO_2_ incubator at 37 °C.The KCNQ4 overexpression plasmid and the control empty plasmid (pcDNA3.1) were transfected into the cells by Lipofectamine2000 transfection reagent.

### Immunohistochemical assay

The collected wax specimens were thinned to a thickness of 3 μm using a microtome. The slices were then transferred and baked at 60 °C. Following this, the slices were immersed in a series of xylene solutions with increasing concentration and sequential alcohol solutions with increasing concentration, including EDTA, for three cycles. The first anti-working solution was added, and the slices were incubated at room temperature. Subsequently, the secondary antibody working solution was added, and incubation at room temperature was done. After adding the DAB chromogenic agent, the color development was observed under an inverted microscope, and the reaction was stopped when the desired color was achieved. The nuclei were restained with hematoxylin dye. The slices were dehydrated with gradient alcohol three times, made transparent with xylene, and finally sealed with neutral gum.

### Cell growth and proliferation assay

For the determination of EdU, the MDA-MB-231 and MDA-MB-468 cells were transfected and then seeded in a 24-well plate. They were incubated with an EdU working solution and subsequently fixed with 4% paraformaldehyde. After that, the cells were permeabilized with 0.2% Triton and stained using the EdU cell proliferation detection kit (Biyun, Shanghai, China). The cells were also stained with DAPI and visualized under a fluorescence microscope. Random visual fields were selected for image capture, and the cell proliferation rate was calculated based on these images.

To perform the MTT assay, the cells were transfected and then seeded into a 96-well plate at a density of 2 × 10^3^ cells per well. After incubation and adhesion, MTT reagent and dimethyl sulfoxide (DMSO) were added to dissolve the crystal. The absorbance of OD 490 nm in each well was measured using an enzyme-linked immunosorbent assay (ELISA).Cell migration and invasion assay. Twenty-four hours post-transfection, the cells were suspended in serum-free medium and transferred to a Transwell chamber (Corning, Cambridge, MA, USA) at a concentration of 3 × 10^4^ cells in 300 μl. Then, 600 μl of medium containing 10% fetal bovine serum was added to the outer side of the chamber. After incubating for 24 h, the chamber was collected, and the number of cells that passed through was counted using Giemsa staining. For the wound healing assay, the transfected cells were seeded in a 6-well plate and allowed to reach 80–90% confluence. A scratch was made in the cell monolayer, and the width of the scratch area was photographed at 0 h and 24 h. The migration rate at each time point was calculated, and a curve was plotted. For the invasion experiments, Transwell chambers were pre-coated with Matrigel matrix (BD Biosciences, San Jose, CA, USA).

### Apoptosis assay

After transfection, the cells were digested with trypsin and rinsed twice with PBS re-suspension. 1 × 10^6^ cells were resuscitated with 100 μl 1 × binding buffer. The control group was divided into blank control group (without staining), 5 μl PE group, 5 μl 7-AAD group and 5 μl PE + 5 μl 7-AAD group. The experimental group was 5 μl PE + 5 μl 7-AAD group. Incubate without light after adding dye solution. Add 1 × binding buffer to each tube and collect it into the flow tube and test it on the computer.

### Statistical analysis

The experimental data were analyzed and visualized using GraphPad Prism 8 and SPSS Statistics 26 software. The Student's t-test is used to evaluate the differences in measurement data between two groups with homogeneity of variance and normal distribution. Spearman correlation test was conducted for correlation analysis. The results showed statistical significance with a threshold of *P* < 0.05.

### Gene expression level analysis

We obtained a comprehensive and standardized pan-cancer dataset called TCGA Pan-Cancer (PANCAN, N = 10,535, G = 60,499) from the UCSC database (https://xenabrowser.net/). we specifically extracted the expression data of ENSG00000117013 (KCNQ4) gene in each dataset. The samples of TCGA were further categorized into Solid Tissue Normal, Primary Tumor, and Primary Blood Derived Cancer-Peripheral Blood. Then we performed log2 (x = 0.001) transformation on each expression value. Subsequently, we excluded the cancer types with less than 3 samples in a single cancer types, and finally obtained the expression data of 26 cancer classes. In order to investigate the expression of KCNQ4 in different cancers, we utilized the tumor immune estimation resource version 2 (TIMER2.0) (http://timer.cistrome.org/) ^12^ "Gene DE" module to extract pan-cancer datasets and associated clinical information. Due to the small amount of normal samples paracancerous and blood) in the TCGA database, so additional data was obtained such as Primary Solid Tumor, Normal Tissue, Primary Blood Derived Cancer-Bone Marrow of normal tissue transcriptome sequencing data, then we distinguished normal samples and tumor samples according to clinical data and analyzed the difference. Finally obtained the expression data of 34 cancer species. The expression profiles of KCNQ4 in different cancers and paired normal cell lines were analyzed using NCI60 on U133A, gcrma and GeneAtlas U133A of BioGPS datasets (http://www.biogps.org) as well as gcrma datasets.

### Cancer staging analysis

Gene Expression Profiling Interactive Analysis (GEPIA, http://www.gepia.cancer-pku.cn/) ^13^ is an interactive web application based on 9736 tumors and 8587 normal samples from TCGA and GTEx databases. It contains the result of a standard processing pipeline for RNA sequencing data. According to the World Health Organization (WHO) classification, the tumors in this dataset are categorized into stages I, II, III, and IV based on Tumor Node Metastasis (TNM) staging. The stage of a tumor indicates the extent of tumor progression, and a higher stage is associated with a poorer prognosis. To investigate the expression of KCNQ4 across different cancer stages in TCGA dataset, we utilized GEPIA and compared the results with the corresponding TCGA normal and GTEx data.

### DNA methylation analysis of KCNQ4

The Shiny Methylation Analysis Resource Tool (SMART) (http://www.bioinfo-zs.com/smartapp/)^14^ is an online database that integrates multi-group and clinical data with DNA methylation to facilitate a comprehensive analysis of promoter methylation status. In this study, SMART was utilized to analyze the methylation level of KCNQ4 in the TCGA database. MethSurv (https://www.biit.cs.ut.ee/methsurv/)^15^ is a web-based tool specifically designed for survival analysis based on CpG methylation patterns It employs methylation data from 7358 different human cancers obtained from the TCGA dataset. For our analysis, the MethSurv tool was used to evaluate the KCNQ4 methylation in the TCGA-BRCA, TCGA-COAD, TCGA-LUAD, and TCGA-UCSC cohorts. Subsequently, survival analysis was conducted based on the methylation status of KCNQ4 at multiple sites.

### Genetic alteration analysis

The cBioportal database (https://www.cbioportal.org/)^16^ is a comprehensive platform that integrates research data from different tumor genomes, enabling the visualization and analysis of operational assumptions. Through this database, we selected the "TCGA Pan-Cancer Atlas Studies" as the cohort for analysis, and analyzed the protein structure, mutation type, mutation site information, copy number change (CAN), and the three-dimensional structure. To specifically study the KCNQ4 gene, we entered it into the "Query" module. Detailed information on alteration sites, types, and numbers for KCNQ4 can be found in the “cancer type summary” and “mutation” modules.

### Analysis of survival and prognosis

The comprehensive and standardized pan-cancer dataset was obtained from the UCSC database to assess the prognosis of KCNQ4. The prognostic indicators utilized included Overall survival (OS), Disease-specific survival (DSS), Disease-free interval (DFS), and Progression-free interval (PFS). The Kaplan–Meier plotter (http://kmplot.com/analysis/)^17^ provides data from GEO, EGA and TCGA databases, enabling the evaluation of gene expression's correlation with patient survival in over 30,000 samples from 21 different tumors. This allows the identification and validation of survival-related biomarkers. The Kaplan–Meier plotter was utilized for analyzing the survival and prognosis of KCNQ4 in various cancer types, with OS serving as the prognostic index. Additionally, the PrognoScan database (http://www.abren.net/PrognoScan/)^18^ was used to reassess the correlation between KCNQ4 expression and breast cancer survival. The threshold value was adjusted to P value < 0.05, and the prognostic index were OS, DMFS and RFS.

### Immune infiltration analysis

Algorithms including Estimate, TIMER, CIBERSOR, EPIC, IPS, MCPcounter, Xcell, CIBERSORT, and QUANTISEQ^19, 20^ were employed to investigate the correlation between KCNQ4 and immune cell infiltration. KCNQ4 expression data were obtained from UCSC, and samples from TCGA-LAML (Primary Blood Derived Cancer-Peripheral Blood), TCGA-SKCM (Metastatic Primary Tumor), Primary Blood Derived Cancer-Bone Marrow, Primary Solid Tumor, and Recurrent Blood Derived Cancer-Bone Marrow were screened. Then the expression profile was mapped onto GeneSymbol. The R software package (version 0.99.9) was utilized to determine the immune cell infiltration score of each patient in each tumor and identify significantly correlated immune infiltration scores. In addition, different algorithms were employed to explore the potential correlation between the expression level of the KCNQ4 gene and the infiltration level of cancer-associated fibroblasts across all cancer types.

### Immune checkpoint analysis

Expression data of KCNQ4 and 60 marker genes belonging two immune checkpoint pathways (Inhibitory(24), Stimulatory(36)) was obtained from UCSC. Then we calculated Pearson correlation coefficients between KCNQ4 and the marked genes of five immune pathways, extracted from a published literature^21^.

### STEMNESS index analysis

Expression data for KCNQ4 was extracted using UCSC. Tumor stemness score was calculated by methylation signature including DNAss and RNAss. We integrated the STEMNESS index, gene expression data of the samples, and excluded samples with less than 3 in any single cancer type. We ultimately got expression data for 37 different cancer types. Subsequently, we calculated the Spearman correlation coefficient by intersecting KCNQ4 expression data with DNAss and RNAss. The visualization of the lollipop plot was performed using the ggplot2 R package, as described in a published literature^22^.

### TMB and MSI analysis

The UCSC database was used to extract expression data of KCNQ4 gene. Samples from Primary Blood Derived Cancer—Peripheral Blood and Primary Tumor were selected for analysis. The maftools R package (version 2.8.05) was utilized to calculate Tumor Mutational Burden(TMB) and Microsatellite Instability (MSI) scores, and it were extracted from a published article^23^.

Protein network and related gene enrichment analysis.

GENEMANIA (http:/genemania.org/)^24^ is a software for predicting gene–gene interactions that enables the generation of hypotheses about gene function. It can analyze gene lists and prioritize genes for functional determination. STRING (https://string-db.org/)^25^ integrates both known and predicted associations between proteins, encompassing physical interactions and functional association. DAVID (https://david.ncifcrf.gov/) offers systematic and comprehensive biological function annotation information for large-scale gene and protein lists, assisting users in extracting valuable biological information. GEPIA was utilized to evaluate the association between KCNQ4 and the selected target genes. Using GENEMANIA database, we identified genes with similar functions to KCNQ4 based on genomics and proteomics data. We downloaded interacting proteins from STRING, and the results of Gene Ontology(GO), including Biological Process(BP), Cellular Component(CC) and Molecular Function(MF). KEGG enrichment analysis^26^ was performed using the official website of DAVID. The protein–protein interaction(PPI) network of KCNQ4 was visualized using the CytoHCA plug-in of the Cytoscape^27^ software application. In addition, GEPIA was employed to analyze KCNQ4 and the first quarter genes including GNAQ, AKT1,KCNJ14, JAK2, GNAO1, CREB1, MAPK3, PRKACA, MAPK1, PRKACB, AKT3, PRKCA, AKT2, KRAS, HRAS, and ADCY6 in the PPI network.

### Prediction of potential drug targets for KCNQ4

Connectivity Map (CMap) (https://clue.io/query)^28^ is an online analysis platform for predicting disease-specific small molecule compounds based on genome-wide transcript expression database. We used CMap to predict potential drug targets for KCNQ4,the list of upregulated and downregulated differentially expressed genes obtained from data analysis was compared with the reference dataset in the database by CMAP. Correlation scores were based on the enrichment of differentially expressed genes in the reference gene expression profile. Statistical significance was set as *P* < 0.05, enrichment scores ranged from -100 to 100 and the results were selected based on the magnitude of the correlation coefficient score to select negatively correlated small molecule compounds. A positive number indicates that the differentially expressed genes upregulated and downregulated are similar to the reference gene expression profile, and negative numbers indicate that the differentially expressed genes upregulated and downregulated may be opposite to the reference gene expression profile. After obtaining the results of CMap analysis, the compounds were selected compounds with the mean coefficient less than minus 95, and were ranked according to their correlation scores.

## Result

### Expression analysis of KCNQ4 mRNA

**Figure 1:**
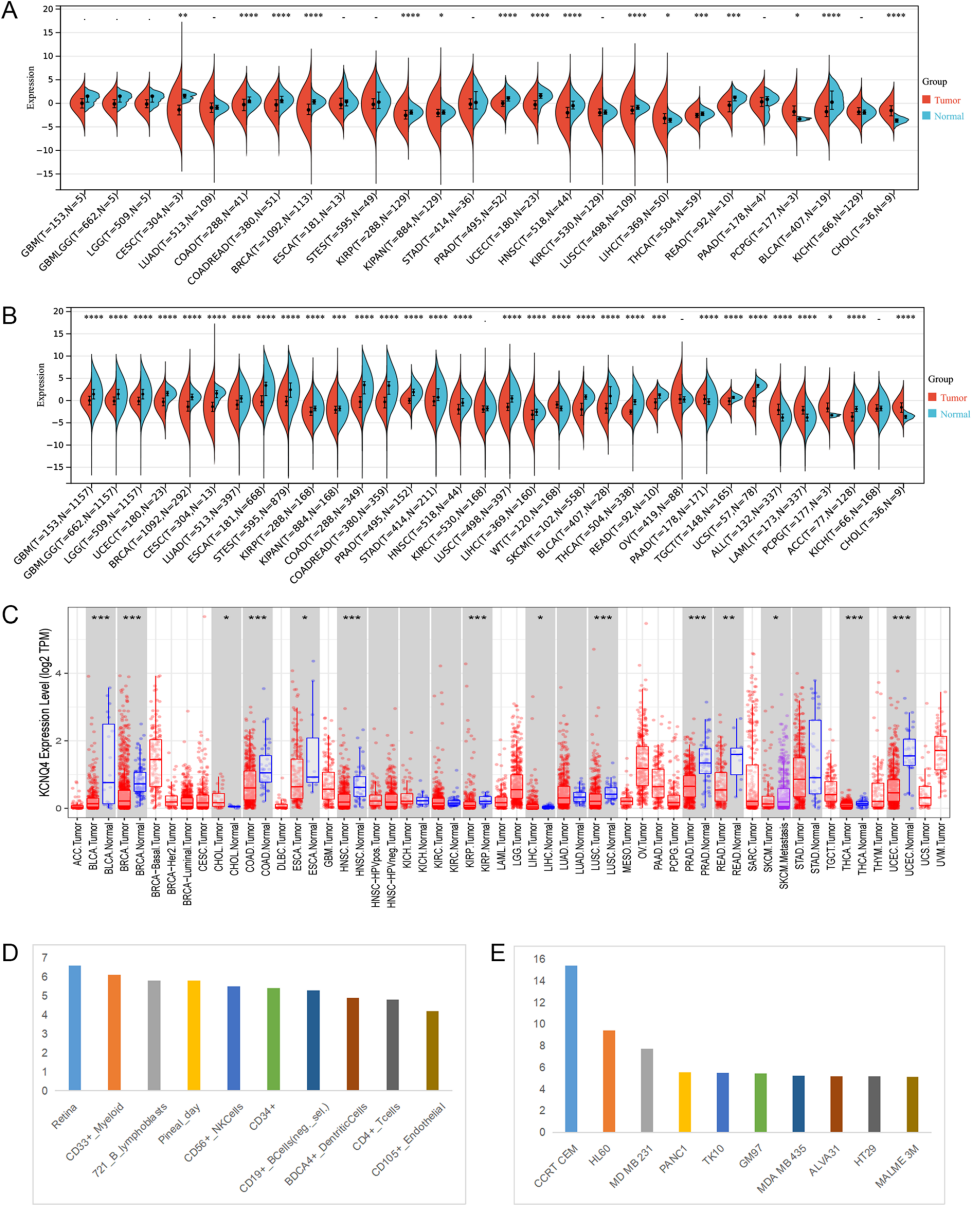
Differential expression of KCNQ4 in different databases. (**A**) KCNQ4 in TCGA dataset. (**B**) The expression level of KCNQ4 in TCGA dataset and the GTEx. (**C**) The expression level of KCNQ4 in TIMER dataset. (**D**) The expression level of KCNQ4 in immune cells via BioGPS database. (**E**) The expression level of KCNQ4 in tumor cells via BioGPS database. *P < 0.05, **P < 0.01, ***P < 0.001.

To investigate the association of KCNQ4 with malignancy, first, we excluded the cancer types with fewer than three samples. Then we analyzed the mRNA expression levels of KCNQ4 in various tumors and normal tissues using different datasets. In TCGA database, compared with normal tissues, KCNQ4 was significantly up-regulated in tumor tissues in three cancers, including LIHC, PCPG and CHOL, while it was significantly down-regulated in 13 cancers, including CESC, COAD, READ, BRCA, KIRP, KIPAN, PRAD, UCEC, HNSC, LUSC, THCA, READ and BLCA (Fig. 1A). We also utilized the TIMER 2.0 database to verify the expression of KCNQ4 in different cancers (Fig. 1C), and the results were consistent with TCGA. However, due to the absence of RNA-seq data of normal tissues in TCGA, we extracted normal tissue data from the GTEX dataset and compared it with TCGA cancer data. The result showed that KCNQ4 was significantly up-regulated in 6 cancers, including WT, PAAD, ALL, LAML, PCPG and CHOL, and significantly down-regulated in 25 cancers, such as GBM, GBMLGG, LGG, UCEC, BRCA and CESC (Fig. 1B). We verified cancer types with different analysis results in different databases by additional databases, and finally decided to use the results from TCGA and GETx as the reference basis for subsequent analysis (Supplementary Fig. 2).Overall, KCNQ4 demonstrated low expression in the majority of cancer types based on data from TCGA and GTEx analyses. Additionally, through the BioGPS database, we observed that KCNQ4 exhibited a low expression level in nearly all cancer cell lines (Supplementary Fig. 3). Among normal cells, immune cells exhibited the highest expression level of KCNQ4 (Fig. 1D). We presented 10 cancer cell lines with the highest KCNQ4 expression level (Fig. 1E). These findings suggest that the expression of KCNQ4 is generally low in tumor tissues and may be involved in the immune regulation process.

### Expression analysis of KCNQ4 cancer staging in different cancer types

**Figure 2:**
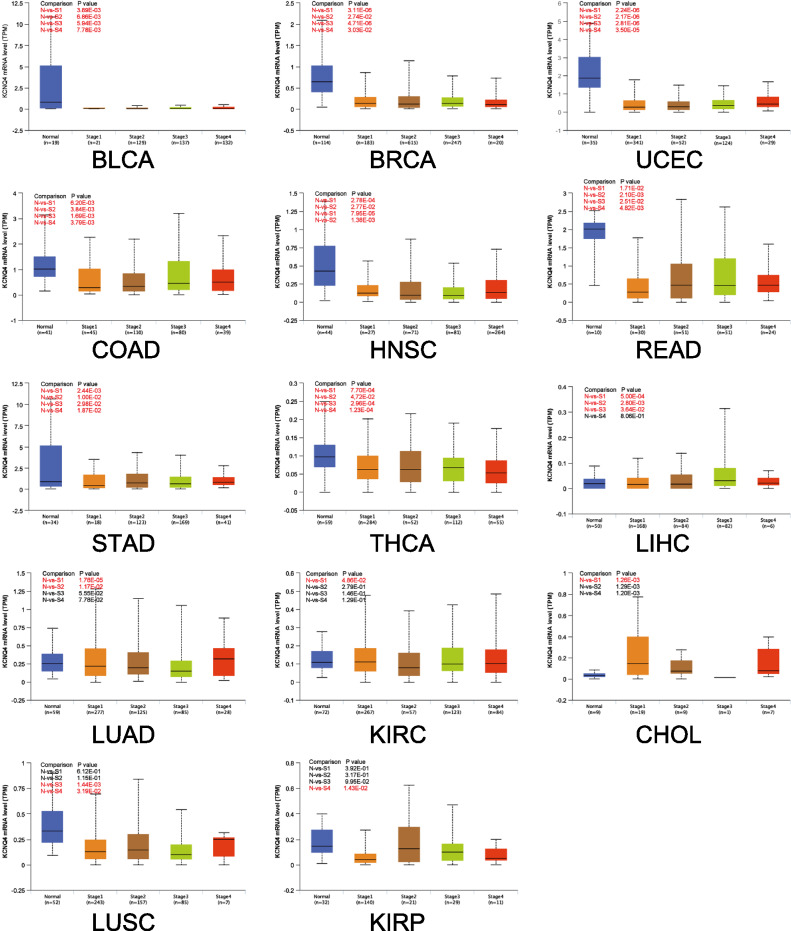
Cancer staging analysis of KCNQ4.The expression of TCGA cancer types in the UALCA database according to the pathological stages of patients . X-axis: cancer staging versus number of samples per stage. Y-axis: transcripts per million. N, normal; S, stage. *P*-values marked in red indicate that both groups are statistically significant.

Next, to investigate the relationship between KCNQ4 expression and tumor development, we analyzed the expression of KCNQ4 in patients with TCGA cancer types, based on their respective cancer stages. Our analysis revealed that the expression of KCNQ4 was lower in BLCA, BRCA, UCEC, COAD, HNSC, READ, STAD and THCA compared to normal tissues. Additionally, we observed that the expression of KCNQ4 was low only in the early stage for LIHC, LUAD, KIRC, and CHOL, while in LUSC and KIRP, it was reduced only in the late stage. These findings show that KCNQ4 may be involved in tumorigenesis in specific cancer types, rather than cancer progression. Interestingly, We found that low expression of KCNQ4 in the above cancers was positively correlated with the low expression in cancer staging (Fig. 2).

### DNA methylation profile of KCNQ4 in different cancers

**Figure 3:**
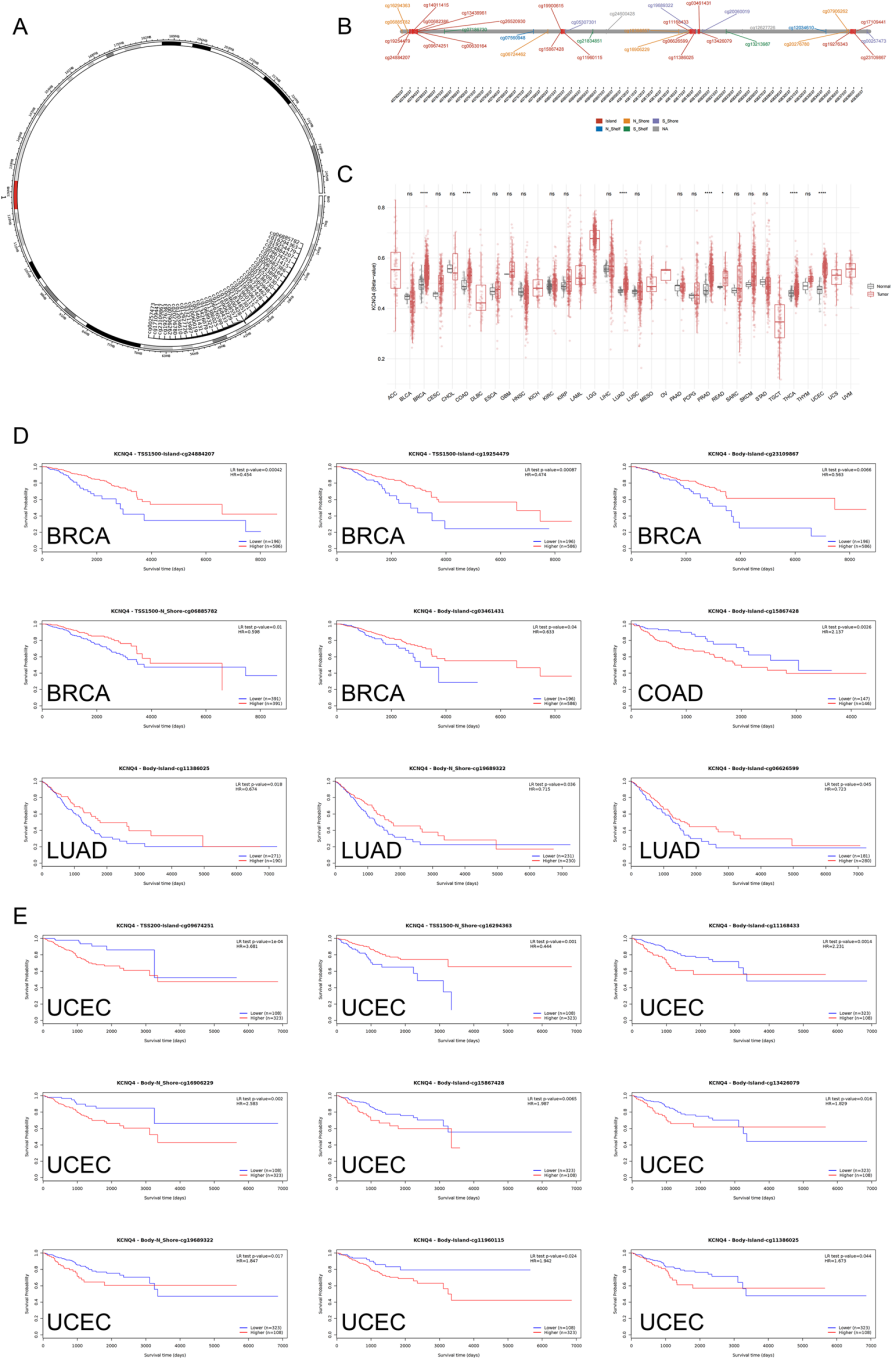
DNA methylation analysis of KCNQ4. (**A**) Chromosomal distribution of the 37 methylation probes associated with KCNQ4 on chromosome 1. (**B**) Detailed location of 37 probes in CpG Island. (**C**) KCNQ4 methylation levels in tumor and normal samples cross different types of cancers. (**D**) KCNQ4 hypomethylation in BRCA, COAD and LUAD has a good prognosis. (E) KCNQ4 hypermethylation in UCEC has a poor prognosis. *p < 0.05; **p < 0.01; ***p < 0.001; ****p < 0.0001.Abbreviation: Ns, no significance.

The hypermethylated DNA promoter suppresses gene expression, whereas hypomethylation activates its expression^13^ Therefore, to elucidate the differential expression of KCNQ4 in various tumors, we examined its methylation status. We used SMART analysis to identify 37 KCNQ4-related methylation probes on chromosome 1, comprising 19 island regions (cg24884207, cg19254479, cg00682386, cg13438961, cg14011415, cg09674251, cg26520930, cg00630164, cg19900615, cg15867428, cg11960115, cg11168433, cg06626599, cg11386025, cg13426079, cg03461431, cg03461431, cg23109867, cg17109441), 7 N Shore regions (cg06885782, cg16294363, cg06724462, cg18398557, cg16906229, cg20276780, and cg07906262), 3 S Shelf regions (cg07186730, cg21834851, and cg13213987), 2 N Shelf regions (cg07560948 and cg07560948), 4 S Shore regions (cg05307301, cg19689322, cg19689322, and cg00257473) and 2 unrecognized regions (cg24600428 and cg12627726) (Fig. 3B). The results from SMART analysis revealed differential methylation patterns of the KCNQ4 gene across various cancers, suggesting its potential functional implications. KCNQ4 methylation levels were significantly lower in BRCA, COAD, LUAD, PRAD, READ, THCA, and UCEC compared to normal tissues (Fig. 3C). These findings suggest that the reduced expression of KCNQ4 in numerous tumors may be attributed to its hypomethylation (Fig. 3). To further verify whether differential expression of KCNQ4 methylation in BRCA, COAD, LUAD, PRAD, THCA and UCEC affects survival, we used MethSurv tools to visualize the correlation between gene expression and DNA methylation. Survival analysis demonstrated that low methylation of KCNQ4 in BRCA, COAD and LUAD was associated with a higher survival rate (Fig. 3D). Conversely, hypermethylation in UCEC was associated with a lower survival rate (Fig. 3E).

### Genetic alteration analysis of KCNQ4

**Figure 4:**
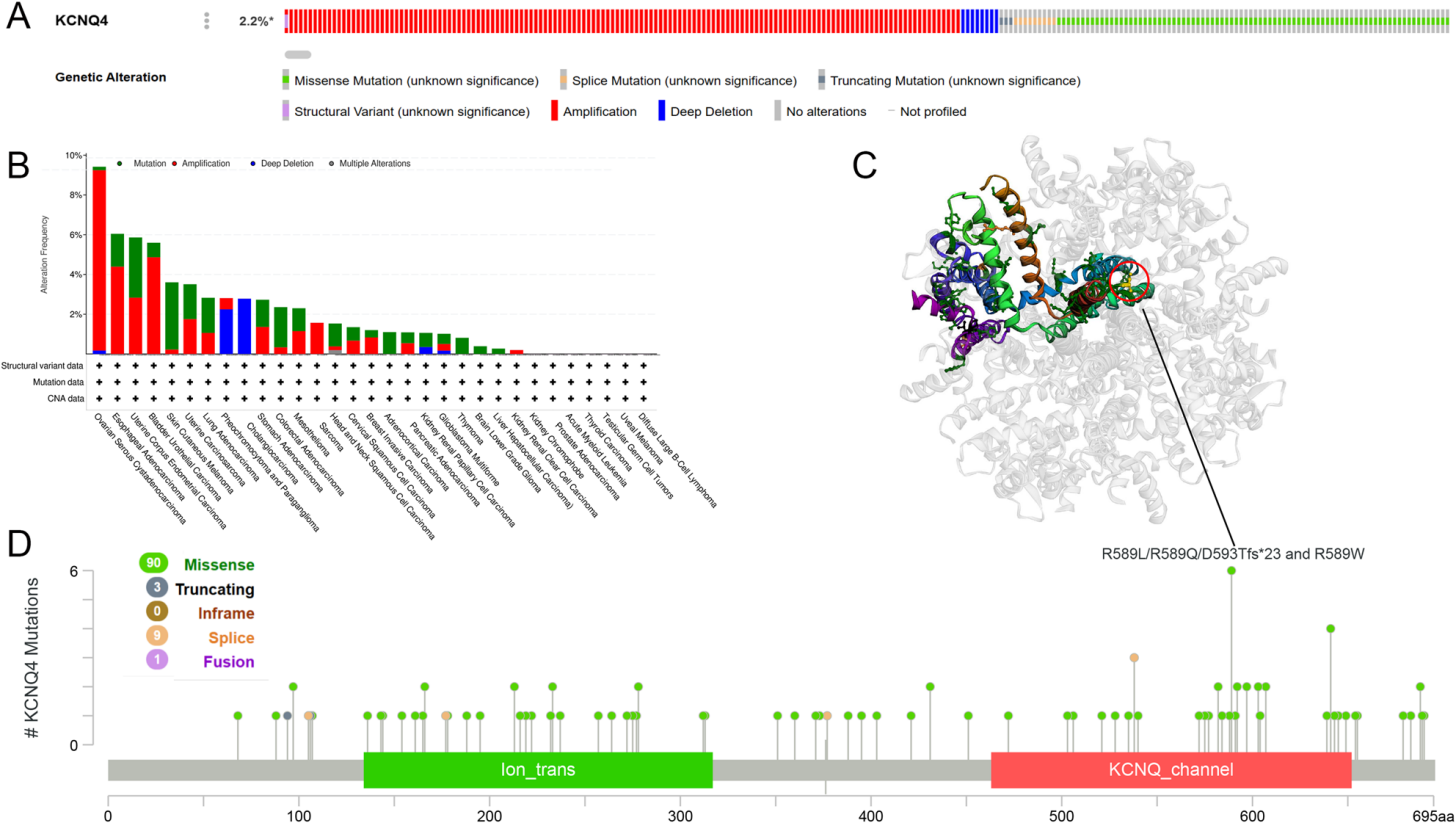
Genetic alteration of KCNQ4 in pan-cancer. (**A**, **B**) The mutation features of KCNQ4 were analyzed by using the cBioPortal. (**C**) The mutation site with the highest alteration frequency (R589L/R589Q/D593Tfs*23 and R589W) in the 3D structure of KCNQ4 via cBioPortal.

The main type of KCNQ4 alteration in different types of cancers was "amplification", followed by “mutation” (Fig. 4A, Supplementary Fig. 8).The frequency of KCNQ4 alterations (> 8%) was highest in OV, where amplification was the predominant type of alteration. Analysis of mutation types and mutation site information for KCNQ4(Fig. 4B) revealed that in BLCA and ESCA cases,where amplification was the primary type of alteration, the frequency of KCNQ4 alteration ranked second and third, respectively, with a prevalence greater than 4%. Among all the cancers, SKCM had the highest frequency of KCNQ4 mutations. In SARC, amplification was the only variant observed, while PCPG exhibited "Deep deletion" as the sole variant form. However, the locations of these genetic alteration were sporadic. For example, the truncation mutation of R589L in the Synaptobrevin domain was exclusively found in two SKCM patients, R589W was observed in only one IDC patient,D593Tfs*23 was only found in one STAD patient and the R589W change was found in only one IDC patient (Fig. 4C, D).

### KCNQ4 was related to patient survival in multiple cancer types

**Figure 5:**
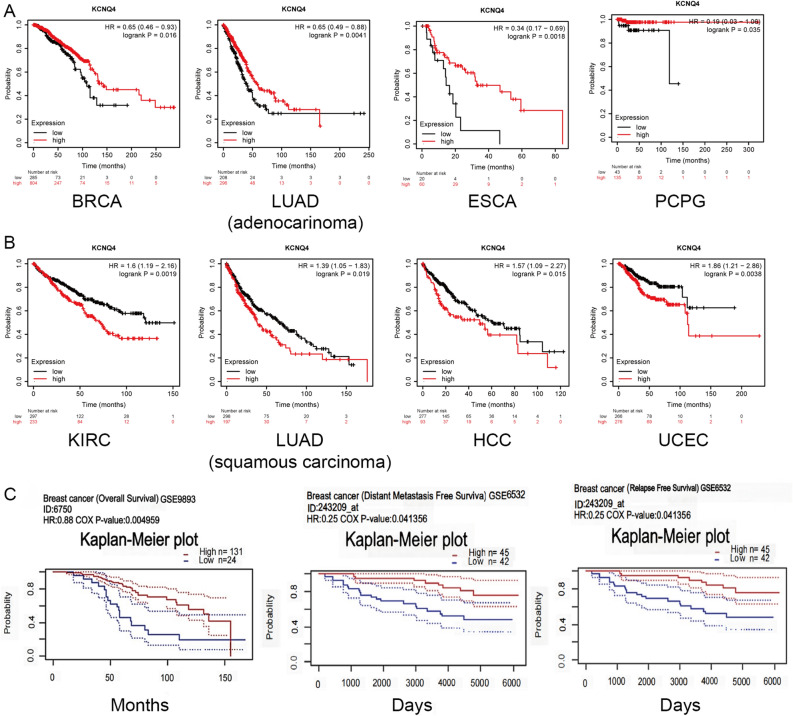
Survival analysis of KCNQ4. (**A**) OS survival curves of cancers with low expression of KCNQ4 associated with a good prognosis via Kaplan–Meier plotter. (**B**) OS survival curves of cancers with high expression of KCNQ4 associated with a poor prognosis via Kaplan–Meier plotter. (C) OS,DMFS and RFS survival curves comparing the high and low expression of KCNQ4 in BRCA patients using PrognoScan database. Data are presented as hazard ratios with 95% confidence intervals and significance was determined by *p* < 0.05.

Next, we utilized UALCAN database to explore the association between KCNQ4 expression and patient outcomes in various types of cancer. High expression of KCNQ4 was found to be correlated with poor OS in GBMLGG, LGG, KIPAN, LIHC, SKCM, SKCM-M, ALL, ACC, and ALL-R. Analysis of the DSS curve revealed a similar trend, with high KCNQ4 expression associated with unfavorable prognosis in GBMLGG, LGG, KIRP, KIPAN, SKCM, and ACC patients. Likewise, the DFS curve demonstrated that high KCNQ4 expression was linked to poorer prognosis in patients with KIRP, KIPAN, and HNSC. Additionally, the PFS curve indicated that high KCNQ4 expression was associated with adverse prognosis in GBMLGG, LGG, KIRP, KIPAN, and ACC patients, suggesting that KCNQ4 may serve as a promising prognostic marker for a variety of cancers (Supplementary Figs. 4, 5, 6, 7). The Kaplan–Meier plotter database incorporates data from TCGA, GEO, and EGA, while the UALCAN database only includes data from TCGA. Furthermore, the KM plotter database offers a larger sample size compared to the UALCAN database, which explains our decision to utilize the KM plotter database and the resulting disparities in our findings. In the "pan-cancer RNA-seq" module, we analyzed the correlation between KCNQ4 expression and OS in different types of cancer. Our analysis revealed that the low expression of KCNQ4 correlated with longer OS in BRCA, LUAD (adenocarcinoma), ESCA, and PCPG (Fig. 5A), while the high expression of KCNQ4 was associated with shorter OS in KIRP, LUSC (squamous carcinoma), HCC, and UCEC patients (Fig. 5B). Notably, KCNQ4 demonstrated favorable prognosis in BRCA (0.37%), ESCA (1.65%), and LUAD (adenocarcinoma) (1.77%) with low mutation frequency, whereas it exhibited poor prognosis in UCEC with high mutation frequency (3.02%). These results suggest that the prognosis impact of KCNQ4 in specific cancers may be influenced by mutation frequency. In addition, we analyzed the survival time of patients with breast cancer, stratified by high or low KCNQ4 expression, using the PrognoScan database in GEO. The results indicated that breast cancer patients with low expression of KCNQ4 had longer OS (*P* = 0.00459, HR = 0.88), DMFS (*P* = 0.041356, HR = 0.25), and RFS (*P* = 0.041356, HR = 0.25) compared to those with high expression of KCNQ4 (Fig. 5C), suggesting a more favorable prognosis. These results also provide valuable insights into the prognosis of KCNQ4 in several specific cancers.

### KCNQ4 was related to the immune infiltration in multiple cancers

**Figure 6:**
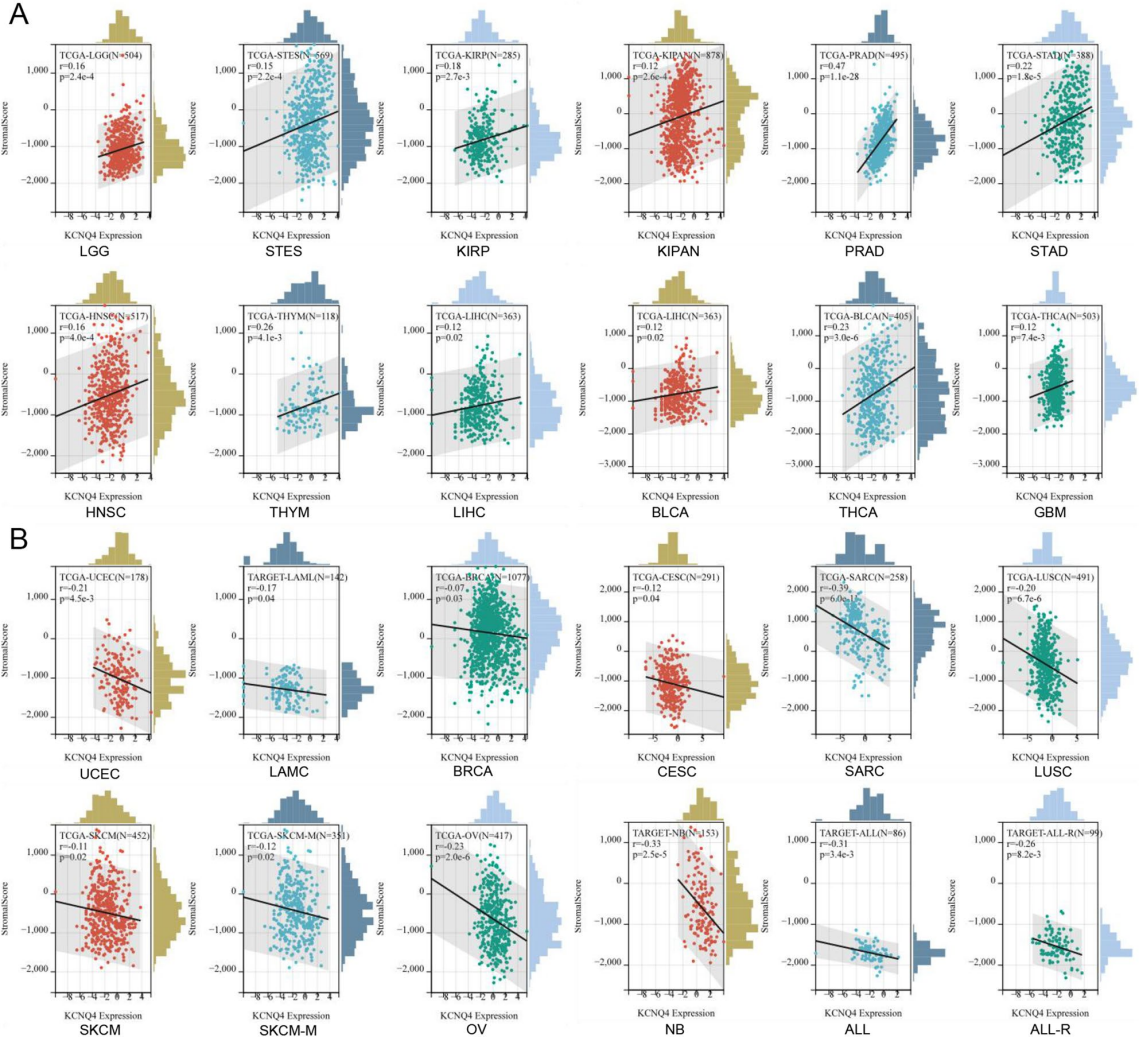
Correlation of KCNQ4 expression with immune infiltration level in pan-cancer via Estimate algorithms. (**A**) Cancer types with positive correlation between KCNQ4 and immune infiltration. (**B**) Cancer types with negative correlation between KCNQ4 and immune infiltration.

**Figure 7:**
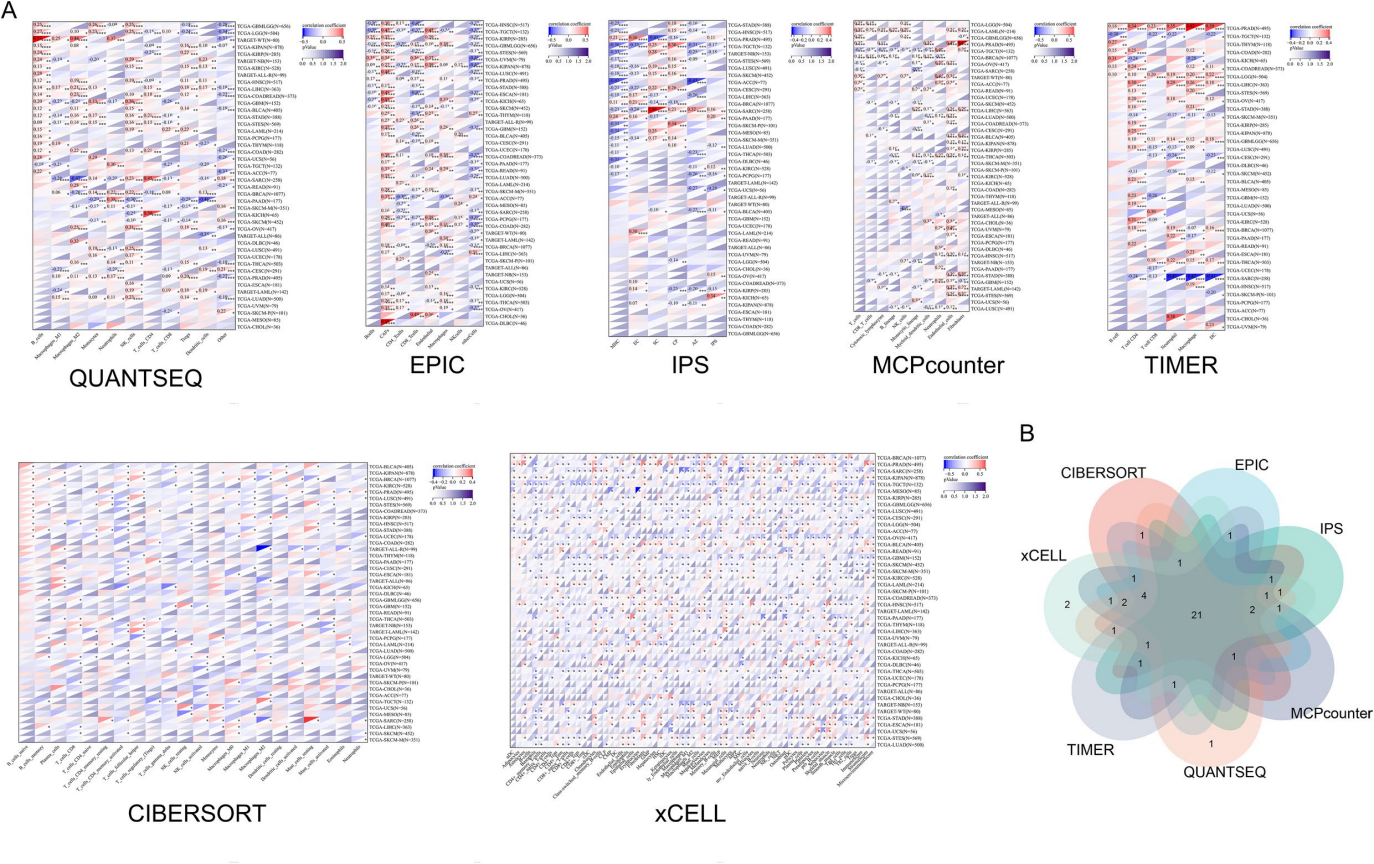
Correlation of KCNQ4 expression with immune infiltration level in pan-cancer via different algorithms. (**A**) QUANTSEQ;EPIC;IPS; MCPcounter; TIMER;CIBERSORT; xCELL. (**B**) Venn diagram of seven algorithmsSeven algorithms take the common cancers after intersection.

**Figure 8:**
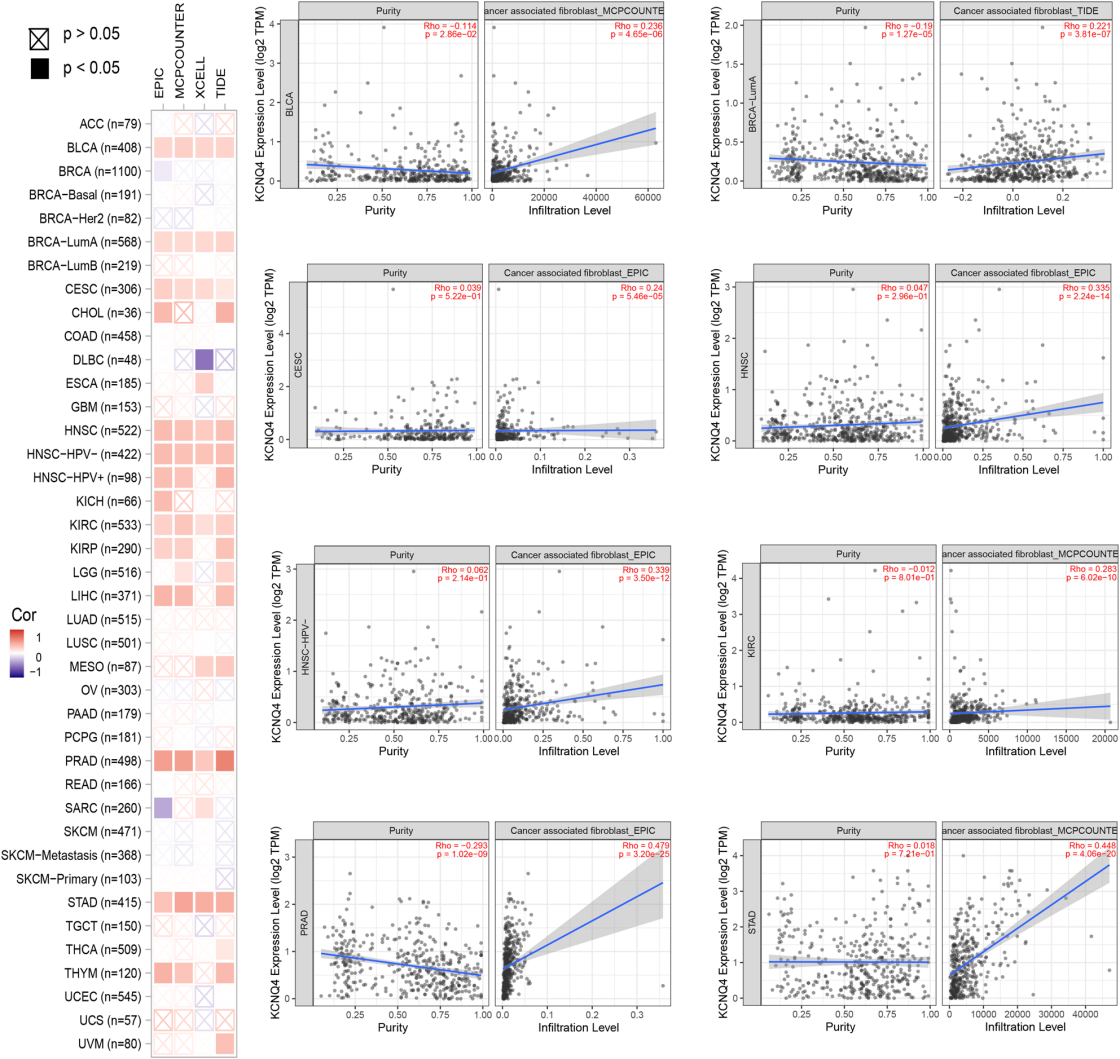
Correlation analysis between KCNQ4 expression and cancer- related fibroblast, which include EPIC, MCPCOUNTER, XCELL, and TIDE algorithms across all TCGA tumors. Red means positive correlation (0–1), blue means negative correlation (− 1 to 0). A value of *p* < 0.05 is considered statistically significant. Correlation values that are not statistically significant are indicated by crosses.

Various methods were employed to analyze the immune infiltration of KCNQ4. The results were then visualized using a Venn diagram. Using the Estimate method, positive correlations were observed between the expression of KCNQ4 and immune infiltration in 12 cancer types, including LGG, STES, KIRP, KIPAN, PRAD, STAD, HNSC, THYM, LIHC, BLCA, THCA and GBM (Fig. 6A). Conversely, in 12 cancer types, namely UCEC, LAML, BRCA, CESC, SARC, LUSC, SKCM, SKCM-M, NB, OV, ALL, and ALL-R, the expression of KCNQ4 exhibited a negative correlation with immune infiltration (Fig. 6B). Through the use of seven methods (CIBERSORT, EPIC, IPS, MCPcounter, QUANTSEQ, TIMER, and xCELL), it was found that KCNQ4 exhibited an association with immunity in multiple cancers (Fig. 7A). We found that the expression of KCNQ4 in 21 types of cancer, including KIPAN, CESC, UCS, TGCT, LUSC, SARC, LIHC, BLCA, LGG, STES, STAD, BRCA, HNSC, PRAD, SKCM, KIRC, THCA, OV, SKCM-M, KIRP, COADREAD, was significantly correlated with immune infiltration (Fig. 7B). In addition, the function of various tumor-infiltrating immune cells (TIICs) is regulated by cancer-associated fibroblasts (CAFs) within the tumor microenvironment (TME). In our study, we discovered a positive correlation between KCNQ4 and tumor-associated fibroblasts in BLCA, BLCA-LumA, CESC, HNSC, HNSC-HPV-, KIRC, PRAD and STAD (Fig. 8A). We generated scatter plot data using an algorithm to depict these tumor associations (Fig. 8B). These findings suggest that KCNQ4 may play a significant biological role in immune infiltration.

### Immune checkpoints gene analysis of KCNQ4

**Figure 9:**
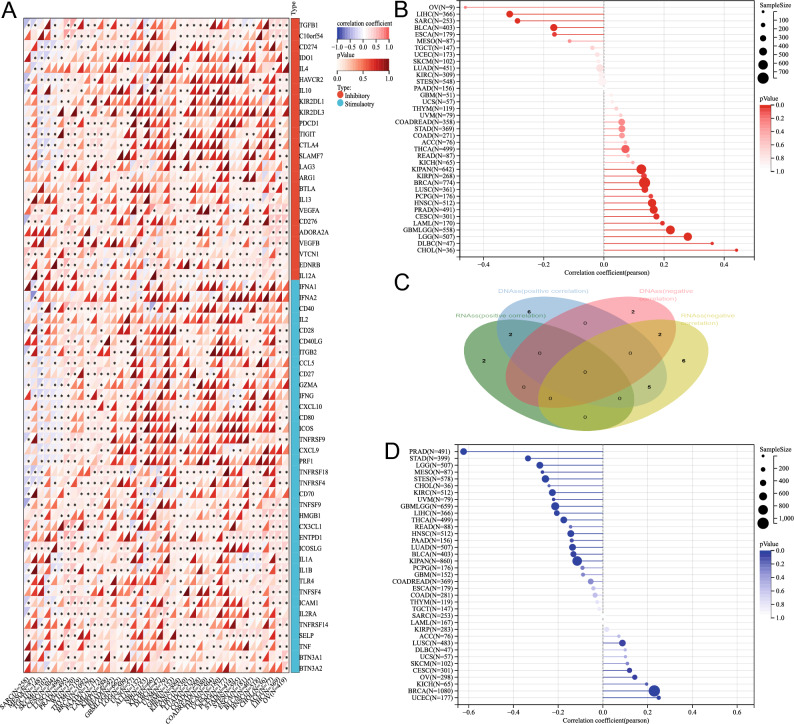
Correlation analysis between KCNQ4 expression with checkpoint and tumor stemness. (**A**) Correlation of KCNQ4 and Immune checkpoint in pan-cancer. (**B**)Association of KCNQ4 and DNAss in pan-cancer. (**C**) Venn diagram of analysis of DNAss and RNAss. (**D**) Correlation of KCNQ4 and RNAss in pan-cancer.

Immunotherapy based on immune checkpoint inhibitors (ICIs) has significantly improved the overall survival of patients with advanced malignant tumors^29^.we conducted correlation analyses to examine the association between the expression of KCNQ4 and immune checkpoint-associated genes in tumors. Our findings indicated a substantial correlation between KCNQ4 expression and the expression levels of immune checkpoint-associated genes in various types of cancer (Fig. 9A).

### Correlation analysis between KCNQ4 and DNAss/RNAss

Cancer stem cells have emerged as crucial targets in cancer research due to their stem-like characteristics, impacting cancer occurrence, treatment resistance, and recurrence. Previous studies have shown that stemness index is related to immune infiltration in the tumor microenvironment, such as DNAss reflects epigenetic characteristics and RNAss reflects gene expression. The higher the stem cell index of these two kinds of tumors, the less the immune cell infiltration in the tumor microenvironment^22^. In this study, we sought to investigate the potential association between the stemness index and immune cells by analyzing KCNQ4 in each tumor via DNAss and RNAss STEMNESS scores based on methylation signature. Our findings revealed a positive correlation between KCNQ4 and GBMLGG, LGG, CESC, LAML, BRCA, KIRP, KIPAN, Prad, HNSC, LUSC, PCPG, CHOL, DLBC, and a negative correlation in ESCA, SARC, LIHC and BLCA via DNAss (Fig. 9B). Additionally, KCNQ4 was positively correlated with CESC, BRCA, UCEC, OV and was negatively correlated in GBMLGG, LGG, LUAD, STES, KIPAN, STAD, PRAD, HNSC, KIRC, LIHC, THCA, MESO, BLCA through RNAss (Fig. 9D). A Venn diagram depicted a positive correlation between KCNQ4 and tumor stemness in CESC and BRCA, while a negative correlation was observed in LIHC and BLCA (Fig. 9C).

### KCNQ4 was related to the TMB and MSI

**Figure 10:**
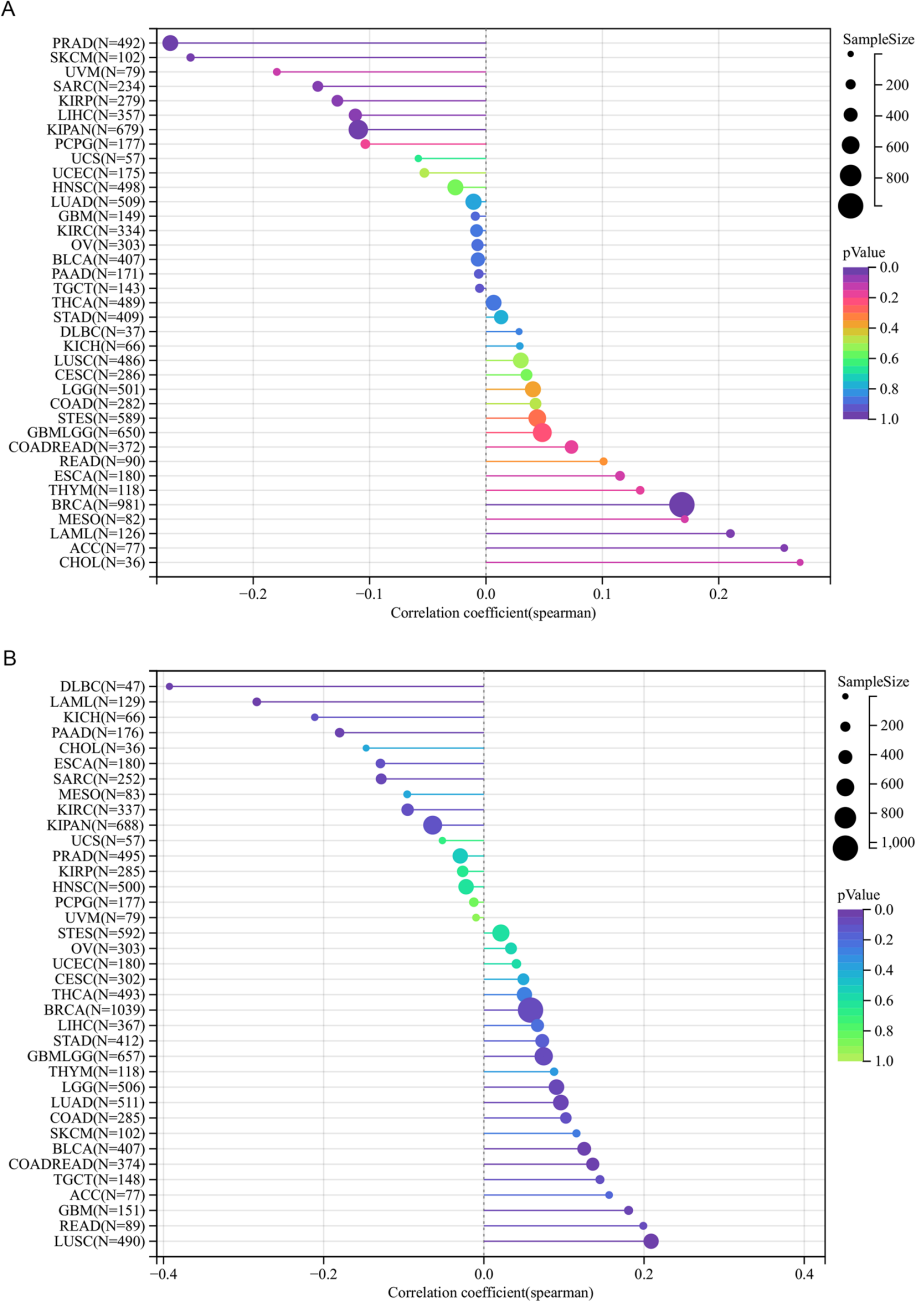
Correlation between KCNQ4 expression with tumor heterogeneity. (**A**) Correlation between KCNQ4 expression with TMB. (**B**) Correlation between KCNQ4 expression with MSI.

In recent years, more and more evidence shows that TMB and MSI can be used as biomarkers to predict immunotherapy response^30, 31^. TMB is a biomarker indicating cancer mutation. MSI is due to the addition or deletion of repetitive units, resulting in microsatellites longer or shorter than normal satellites. A number of clinical studies have confirmed that patients with high TMB and TMB tumors have higher clinical benefits after receiving immune checkpoint inhibitors^32, 33^. So an analysis of the TMB and MSI was conducted on the gene KCNQ4. The results showed a positive correlation between KCNQ4 and ACC and KICH in terms of TMB. Conversely, KCNQ4 demonstrates a negative correlation with KIRP in the same analysis (Fig. 10A). Similarly, the analysis based on MSI revealed a positive correlation between KCNQ4 and several tumor types, namely LGG, LUSC, READ, BLCA, and ACC. On the other hand, KCNQ4 exhibited a negative correlation with LAML, PAAD, and DLBC using the MSI analysis (Fig. 10B). The above results provide reference value for some specific tumors in immunotherapy.

### Enrichment analysis of KCNQ4

**Figure 11:**
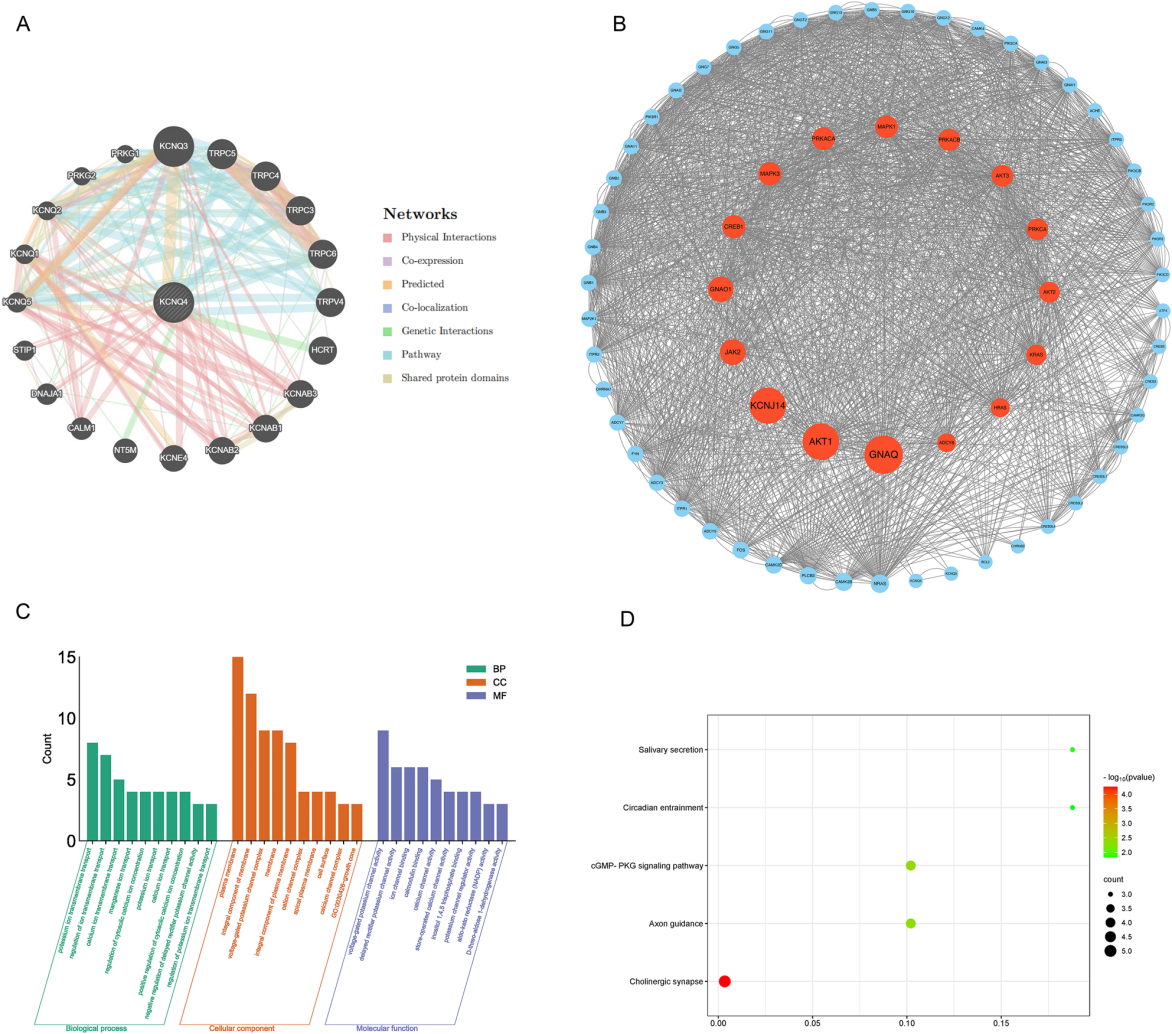
Protein network and enrichment analysis of KCNQ4. (**A**) The potential interaction molecular network of KCNQ4 was created via GENEMANIA. (**B**) Protein network map network of KCNQ4 via cystoscope. (**C**) GO pathway analysis of KCNQ4. (**D**) KEGG pathway analysis of KCNQ4.

**Figure 12:**
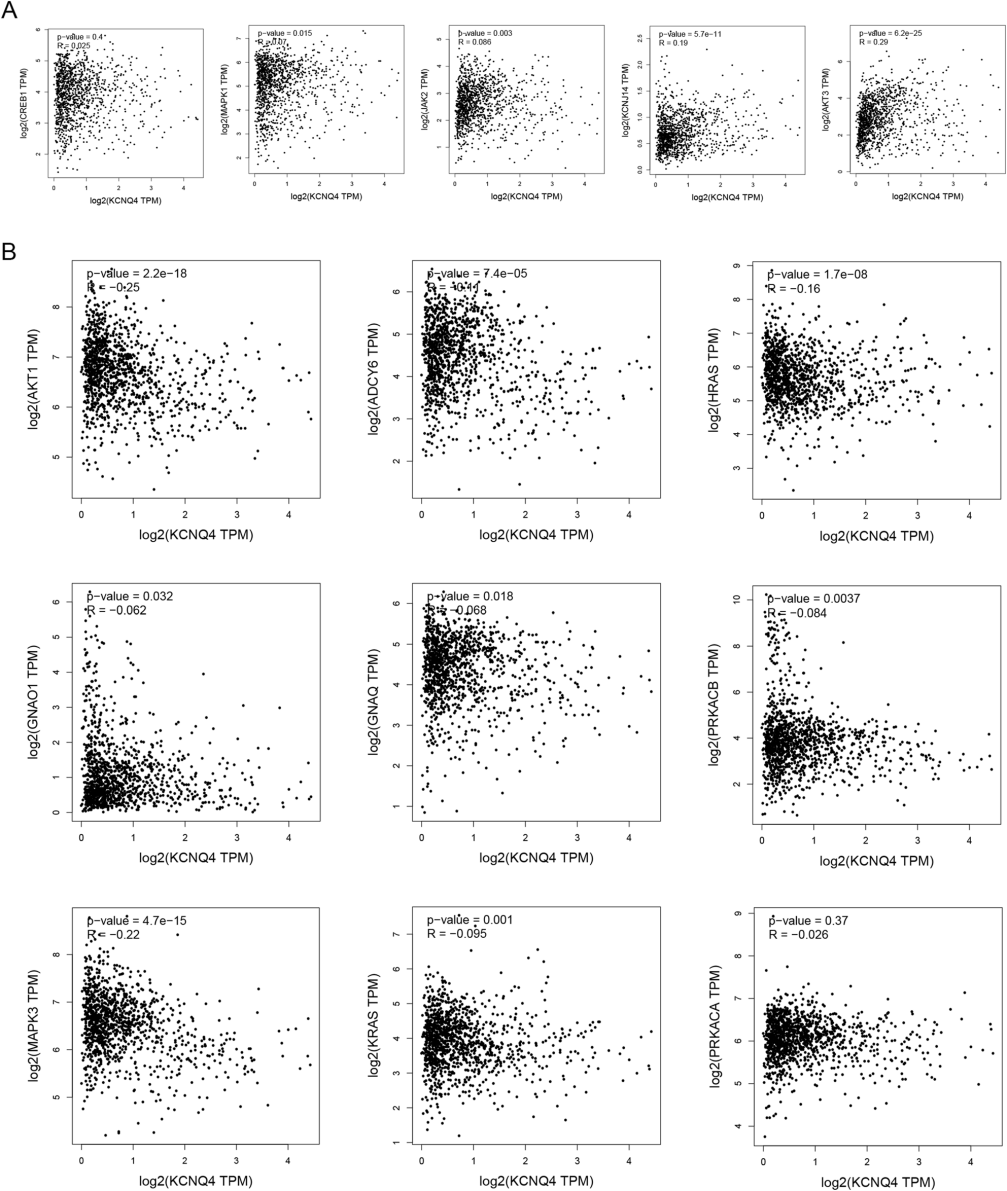
Correlation of KCNQ4 and its related gene in PPI network. (**A**) Genes associated with KCNQ4 positively. (**B**) Genes associated with KCNQ4 negatively.

To further explore the molecular mechanism of KCNQ4 in tumors. We utilized the GENEMINA database to screen for potential interacting genes (Fig. 11A).Then the STRING database was used to download related proteins and subsequently plotted PPI interaction map (Fig. 11B). By conducting GO (Fig. 11C) and KEGG enrichment (Fig. 11D) analysis, using combining the combined set of potential interacting genes mentioned above ,we found these genes are related to the BP , such as potassium transport, regulation of ion transport and calcium transport. The CC terms are associated with the plasma membrane, membrane assembly and voltage-gated potassium channel complex. The MF terms included voltage-gated potassium channel activity, delayed rectifier potassium channel activity and ion channel binding. Further KEGG enrichment analysis showed that these genes were associated with cholinergic synapses, cGMP-PKG signaling pathway, axonal guidance, salivary secretion, and circadian entrainment. We utilized spearman rank correlation of GEPIA to evaluate the positive (Fig. 12A) and negative (Fig. 12B) association in BRCA between KCNQ4 and a subset of target genes.

### Prediction of potential drug targets for KCNQ4 by CMap

**Figure 13:**
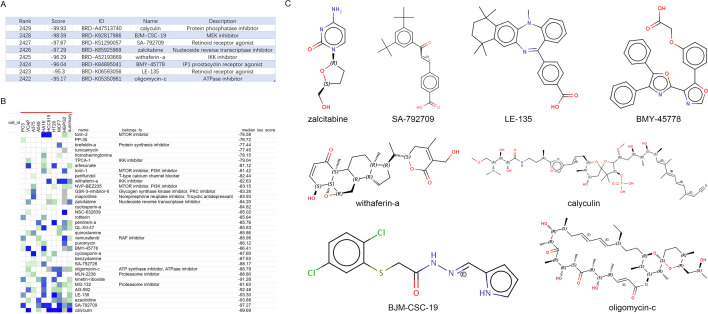
Prediction of potential therapeutic molecular compounds by connectivity maps. (**A**) Compounds with average coefficients less than − 95 were selected and ranked according to their correlation scores. (**B**) A heat map to present the overall data. (**C**) Chemical structures of eight small molecule compounds.Note: PC3, human prostate cancer cell line. VCAP, vertebral carcinoma of the prostate cell line. A375, a human melanoma cell line. A549, adenocarcinoma human alveolar basal epithelial cells. HA1E, a human embryonic kidney cell line. HCC515, a human lung cancer cell line. HT29, a human colorectal adenocarcinoma cell line. MCF7, a breast cancer cell line. HEPG2, a human hepatocellular carcinoma cell line.

The genes, small molecule compounds and diseases are closely interconnected, CMap can provide an important platform for effective anti-cancer drug therapy^34^. According to the requirements of the CMap, the differentially expressed genes in the results were inputted into data in up and down files. Through analysis, the relevant Connectivity values of the compounds were obtained, and a total of 2431 compounds had negative score values. Eight potential compounds were identified, including calyculin, BJM-CSC-19, SA-792709, zalcitabine, withaferin-a, BMY-45778, LE-135 and oligomycin-c (Fig. 13A).We also use the form of a heatmap to present the overall data. The color of the heat map represents the score value, with higher values in red and lower values in gray (Fig. 13B). Finally, we also show the chemical structure formula of small molecule drugs (Fig. 13C).

Expression of KCNQ4 in breast cancer tissue was lower than normal tissue.

**Figure 14:**
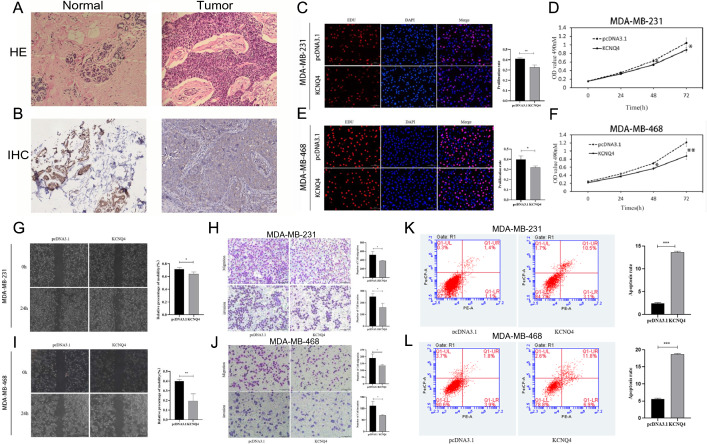
Biological function of KCNQ4 in breast cancer. (**A**) The normal breast tissue and breast tissue by HE staining. (**B**) The normal breast tissue and breast tissue by IHC. (**C**, **D**) The results of EdU assays in MDA-MB-231 after upregulating of KCNQ4. (**E**, **F**) The results of MTT assays in MDA-MB-468 after upregulating of KCNQ4. (**G**, **H**, **K**) The representative micrographs of wound healing, transwell and apoptosis assays in MDA-MB-231 after KCNQ4 overexpression. (**I**, **J**, **L**) The representative micrographs of wound healing, transwell and apoptosis assays in MDA-MB-468 after upregulating of KCNQ4. Error bars represent the S.D. obtained from three independent experiments; *p < 0.05, **p < 0.01, ***p < 0.001.

To verify the relative expression of KCNQ4 in breast cancer, we initially examined the normal breast and breast cancer tissues using HE staining (Fig. 14A), and verified the expression level of KCNQ4 in breast cancer through IHC immunohistochemistry IHC experiment (Fig. 14B). The results showed a significant decrease in KCNQ4 expression in tumor tissue compared to normal tissue.

### Overexpression of KCNQ4 inhibited the proliferation, migration, and invasion of breast cancer

We hypothesized that KCNQ4 as a anticancerous gene in breast cancer. To investigate the effect of KCNQ4 on breast cancer cells proliferation, we conducted EdU and MTT assay. The EdU proliferation assay, the results revealed that breast cancer cells MDA-MB-231(Fig. 14C) and MDA-MB-468 (Fig. 14E) with overexpressed KCNQ4 plasmid exhibited significantly reduced proliferation rates compared to the control group. The MTT proliferation assay indicated that the proliferation rate of breast cancer MDA-MB-231 (Fig. 14D) and MDA-MB-468 (Fig. 14F) cells transfected with KCNQ4 plasmid was significantly lower than that of the control group. Furthermore, we used wound healing and transwell assay to evaluate the impact of KCNQ4 on the migration and invasion of breast cancer cells. The wound healing assay demonstrated that KCNQ4 could inhibit the migration of breast cancer cells MDA-MB-231 (Fig. 14G) and MDA-MB-468 (Fig. 14I). The Transwell assay demonstrated a significant decrease in the number of cells transfected with overexpressed KCNQ4 plasmid through the filter membrane compared to the control group, suggesting the inhibitory effect of KCNQ4 on the migration and invasion of MDA-MB-231 (Fig. 14H) and MDA-MB-468 (Fig. 14J) breast cancer cells.

### Overexpression of KCNQ4 enhanced breast cancer cell apoptosis

Flow cytometry was used to assess the cell cycle distribution of KCNQ4, but no positive results were obtained (Supplementary Fig. 9) Interestingly, the apoptosis assay showed that the overexpression of KCNQ4 promoted apoptosis of MDA-MB-231 (Fig. 14K) and MDA-MB-468 (Fig. 14L) compared to the control.

## Discussion

Cancer is a disease originated from the accumulation of genetically mutated somatic cells in the human body. Nowadays, cancer has become a major global public health concern, despite advances in chemotherapy and immunotherapy, long-term anti-tumor responses are often inefficient, leading to the need for new targets for diagnosis and treatment^35^. According to the results of our pan-cancer analysis, KCNQ4 is aberrantly expressed in a range of malignancies, but until now, most of the research on KCNQ4 has focused on non-syndromic hearing loss and less on cancer^36^. Therefore, a comprehensive and systematic analysis of KCNQ4 in the context of pan-cancer is crucial. We used multiple databases to determine the expression, prognosis and other characteristic of KCNQ4 shows that the expression of KCNQ4 is low expression in most tumor types. The findings are consistent with previous research, indicating that expression of KCNQ4 is low in BRCA and PRAD^8, 11^. Additionally, in 11 cancer types, expression of KCNQ4 was substantially correlated with cancer staging. The aforementioned data suggest that KCNQ4 may play a role in inhibiting tumor by affecting the occurrence and development of tumors. Furthermore, we analyzed the mutation frequency, DNA methylation and prognosis in pan-cancer and tried to find the potential association. A significant finding is in BRCA, LUAD, ESCA with low expression of KCNQ4, the mutation frequency and methylation are also low with good prognosis. On the contrary in UCEC with high expression of KCNQ4, the mutation frequency and methylation are also high with a bad prognosis. By IHC and *vitro* assays, we confirmed that KCNQ4 was low in BRCA and acted as a tumor suppressor. However, the analysis of mRNA, mutation, methylation and prognosis of KCNQ4 shows that the expression of KCNQ4 is different in different tumor types, not only we have confirmed that KCNQ4 can affect the biological behavior of breast cancer cells, but also may affect other unexplored cancers, which needs to be verified by further molecular biology and clinical experiments.

TME and various biological factors bring opportunities and challenges in tumor treatment. One crucial element in this environment is immune infiltration, which significantly influences tumor development and prognosis ^37^. In TME, tumor cells and immune cells regulate each other, in which immune cells are closely related to immune checkpoints and the treatment of cancer^38^. In this study, we also discussed the relationship between the expression of KCNQ4 and immune cells and immune cell checkpoint gene (ICG). The results showed that KCNQ4 was significantly correlated with the score of immune invasion in most tumors. We further discussed the relationship between the expression of KCNQ4 and immune checkpoint gene (ICG) and found that it was related to immune genes. Nowadays, immune checkpoint inhibitors (ICIs) are becoming the most effective immunotherapy for cancer treatment. ICI aims to reverse the immunosuppressive tumor environment by targeting (ICG), thus enhancing the anti-tumor immune response by activating immune infiltrating cells^39^. As the characteristics of different tumors are different, it also needs clinical verification, which also provides a new idea for tumor immunotherapy, and further establishes the value of KCNQ4 in the diagnosis and treatment of tumors. Recent studies have shown the complexity of tumor dryness and immune heterogeneity and their potential for clinical relevance in immunotherapy^40, 41^. Therefore, exploring the mechanism of dryness and heterogeneity of tumor immune microenvironment is helpful for our clinical evaluation of malignant tumors, thus promoting the development of more effective personalized therapy. Our study results on the dryness of KCNQ4, TMB and MSI emphasize the strong correlation between the expression of KCNQ4 and the dryness and heterogeneity of tumor genomes, especially in BRCA, CESC, LIHC, BLCA, which may play a role in improving the status quo of clinical treatment of cancer.

Gene enrichment analysis can effectively reveal the relationship between biological processes and gene expression profiles (RF), and provide a reliable research basis. We carried out GO and KEGG enrichment analysis of KCNQ4-related genes and found that KCNQ4 is enriched in Cholinergic synapse, ion transmembrane transport and other signal pathways, and is closely related to cancer and immunity. Some studies have shown that in the KCNQ4-enriched KEGG pathway, cholinergic and immune system can interact with cancer to evade host immunity^42^, the secretion of axon guide molecule (AGM) can indirectly play an important role in BRCA^43^, cGMP/PKG signal transduction can inhibit tumor immunity^44^, oral dysfunction related to saliva secretion can affect the prognosis of patients with head and neck cancer (HNC)^45^. Patients with low circadian rhythm circadian rhythm signature (CRS) may have a higher TMB and are more likely to benefit from immune checkpoint blocking (ICB) therapy^46^. In the GO pathway enriched by KCNQ4, a large number of studies have shown that the transmembrane transport and concentration of ion channels as transmembrane proteins are also closely related to cancer^47–51^.Recent studies have found that plasma membrane disturbance and tumor cell lysis can be an integral part of multimodal therapy strategies for cancer patients through oncolytic polymers as new targets and killing mechanisms^52^. The glycoprotein interaction on the cell surface can participate in immune escape^53^ .Inositol 1, 4, 5-triphosphate (IP3Rs) acts as a channel for intracellular calcium (Ca) 2 + to release cellular bioenergetics and participates in cell proliferation and death in cancer^53^. Therefore, targeting enriched channels and regulating their activity may provide a new way for the treatment of cancer. These conclusions provide some evidence for the follow-up molecular biology research of KCNQ4 in cancer, and are expected to become an effective method for biological researchers and clinicians to identify and diagnose specific diseases.

Currently, Small molecules as cancer targeting ligands play an important role in the field of anti-tumor and targeting different cancers have shown potential as anticancer treatments^54^. Among the small molecule compounds we screened, Calyculin as an effective protein phosphatase inhibitor has been reported that Combination of celecoxib and Calyculin-A(CLA) inhibited epithelial-mesenchymal transition in human oral cancer cells, CLA can induced apoptosis in human osteosarcoma MG63 cells^55^. Zalcitabine induce neural tube malformation and induce the proliferation and apoptosis of neuroepithelial cells^56^. Withaferin A-induced ROS inhibited hepatocellular carcinoma cell growth and migration through the inhibition of IGF2BP3 to deactivate JAK2/STAT3 signaling^57^, it can also inhibit breast cancer-induced osteoclast differentiation^58^. LE135, a retinoid acid receptor antagonist, produces pain through direct activation of TRP channels^59^. Oligomycins as inhibitors of oncogenic mutant K-Ras membrane localization were considered a potential chemotherapeutic agent for cancer^60^. However, BJM-CSC-19, BMY-45778 and SA-792709 are still being studied. Therefore, they can as an attractive approach to cancer therapy.

Although our comprehensive pan-cancer analysis provides insights into the tumor inhibitory effect of KCNQ4, there are still significant limitations that need to be addressed in the future KCNQ4 analysis of key tumors. First of all, our research is based on bioinformatics and different public databases, there are differences in data sources and analysis results, and the specific mechanism of KCNQ4 expression, mutation or methylation difference in various tumors is not clear. Further, we should focus on the role of KCNQ4 in immune infiltration and treatment. Thirdly, we only carried out immunohistochemical and in vitro functional experiments on KCNQ4 in the breast, which should be further verified in vivo, including animal models. Finally, KCNQ should also be validated in several other related cancers.

## Conclusion

In summary, through bioinformatics analysis, the abnormal expression of KCNQ4 in pan-cancer is closely related to methylation, mutation, prognosis, heterogeneity, dryness, and immune infiltration. Through IHC experiments and cultured cell experiments, it is proved that the decreased expression of KCNQ4 in breast cancer can inhibit proliferation, migration, invasion and promote apoptosis of tumor cells, indicating its potential as a target for cancer therapy.

### Supplementary Information

Supplementary Figures.

## Data Availability

The datasets analyzed during the current study are available in the UCSC database(http://www.genome.ucsc.edu/), GEO (https://www.ncbi.nlm.nih.gov/geo/), TIMER2.0 (http://www.timer.cistrome.org/), cBioportal database (https://www.cbioportal.org/),BioGPS database(http://www.biogps.org/#goto=welcome),GENEMANIA (http://www.genemania.org/), STRING (https://www.string-db.org/) , DAVID (https://www.david.ncifcrf.gov/), GEPIA, http://www.gepia.cancer-pku.cn/), SMART (http://www.bioinfo-zs.com/smartapp/), MethSurv(https://www.biit.cs.ut.ee/methsurv/) , CMap (https://www.clue.io/query), Kaplan–Meier plotter (http://www.kmplot.com/analysis/).
