# Supplementary material for: Comprehensive pan‑cancer analysis of potassium voltage-gated channel Q4 (KCNQ4) gene across multiple human malignant tumors
Source: Sci Rep. 2023 Oct 30;13:18608. doi: 10.1038/s41598-023-45074-7 (PMC10616121; doi:10.1038/s41598-023-45074-7)
Supplement: Supplementary file 1 — Supplementary Figures. [file 41598_2023_45074_MOESM1_ESM.docx]

**Comprehensive pan‑cancer analysis of**

**Potassium Voltage-Gated Channel Q4（KCNQ4 ）gene**

**expression in human malignant tumors**

Qing Zhao^12＋^ Yunxiang Zhang1^*^

*Correspondence:

Yunxiang Zhang

zhangbing199592@163.com

1 Pathology Department, First Affiliated Hospital of Weifang Medical University (Weifang People’s Hospital), Weifang, China

2 Department of Basic Medicine, Weifang Medical University, Weifang, China


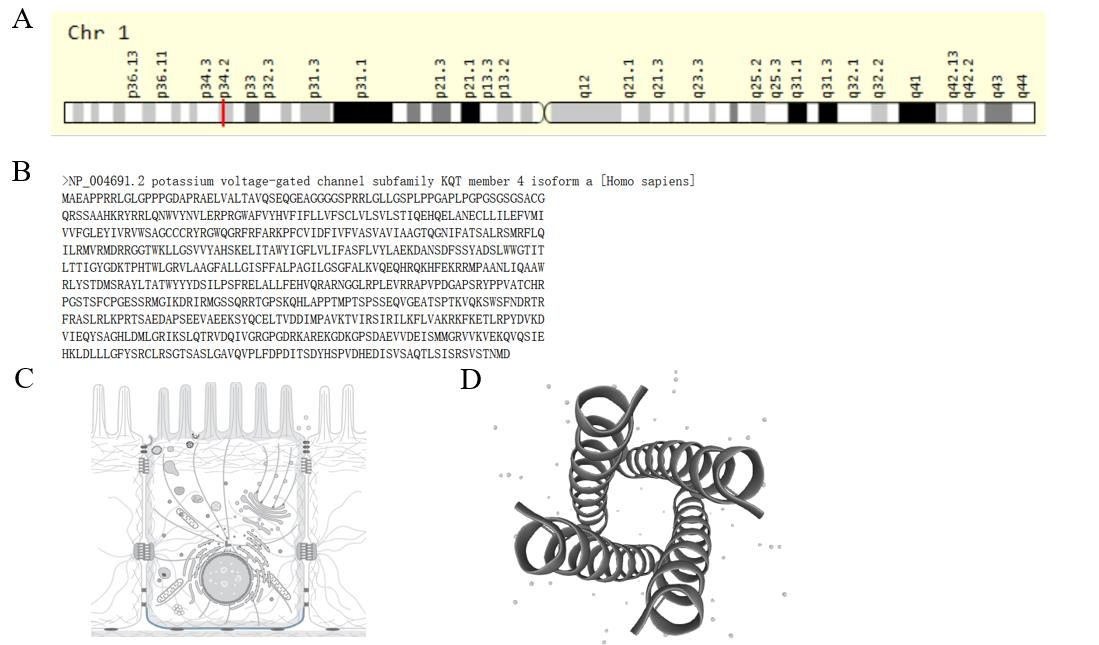
**Supplementary Figure1** |**Genomic and protein features of KCNQ4**

(Supplementary Figure 1A)The genetic characteristics of KCNQ4 were obtained via NCBI(https://www.ncbi.nlm.nih.gov/).(Supplementary Figure1B)The amino acid sequence of KCNQ4 via GeneCards(https://www.genecards.org/).(Supplementary Figure 1C)The location of KCNQ4 via UniProt(https://www.uniprot.org/).(Supplementary Figure 1D)The structure of KCNQ4 via UniProt(https://www.uniprot.org/).


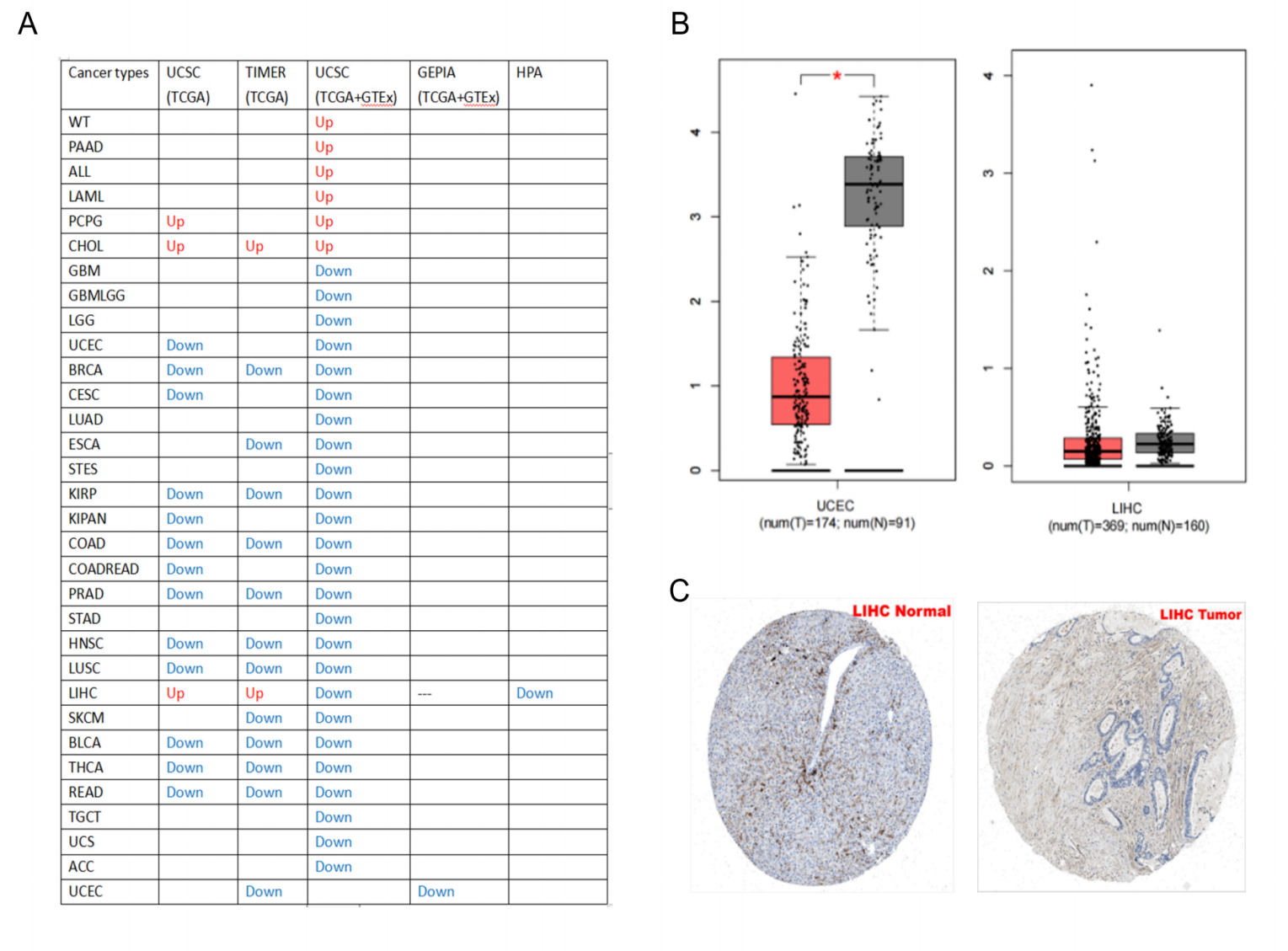


**Supplementary Figure 2 |Expression and validation of KCNQ4 in different databases**

(Supplementary Figure 2A)The expression of KCNQ4 in different cancer types from multiple databases.(Supplementary Figure 2B)The expression of KCNQ4 in UCEC and LIHC via Gene Expression Profiling Interactive Analysis(GEPIA，http://gepia.cancer-pku.cn/）（Supplementary Figure 2C）The protein expression of KCNQ4 in LIHC via[The Human Protein Atlas](https://www.proteinatlas.org/)(HPA,https://www.proteinatlas.org)


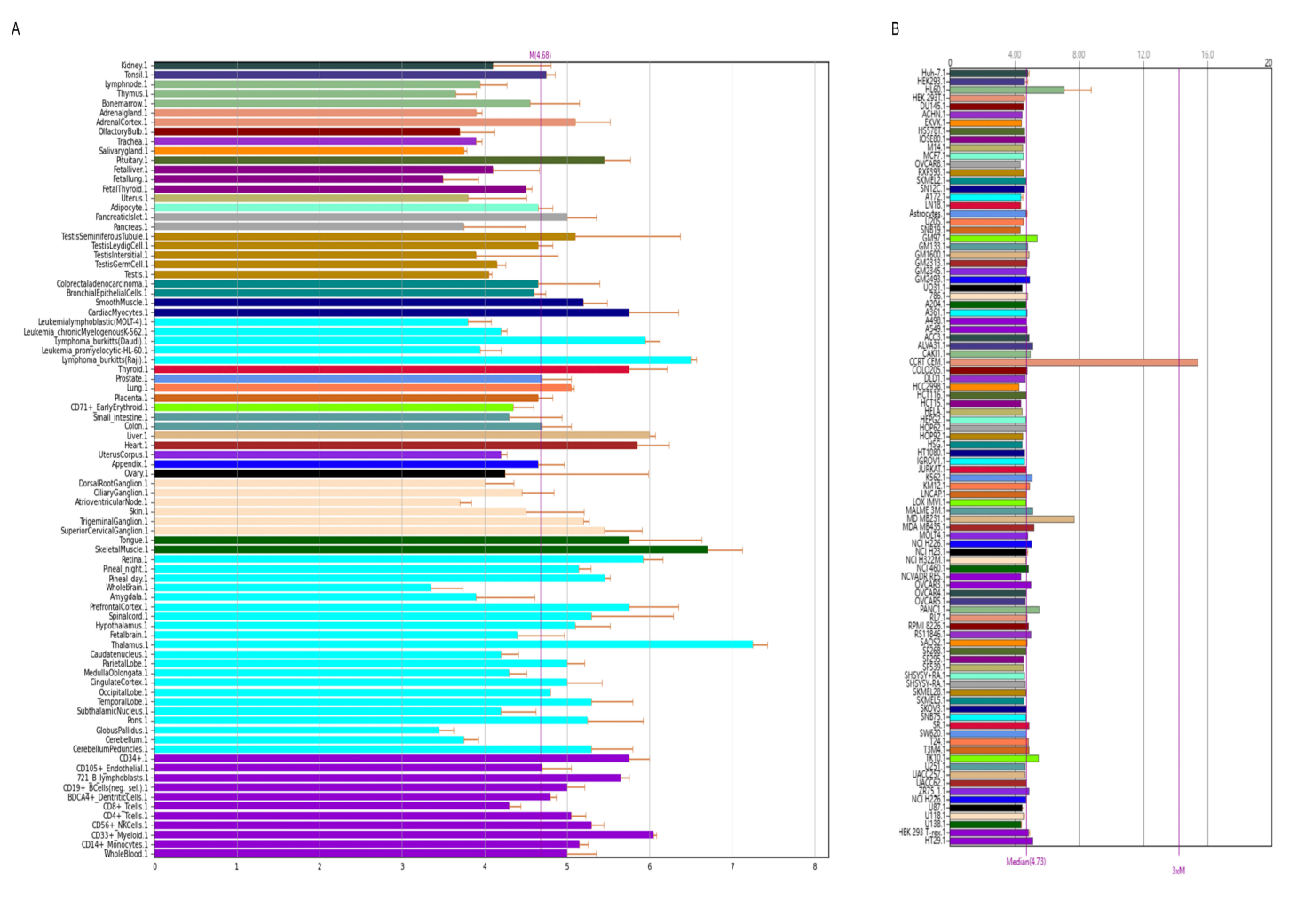
**Supplementary Figure 3** | **The expression level of KCNQ4 in normal tissues and cancer cells via BioGPS**

(Supplementary Figure 3A)The expression level of KCNQ4 in normal tissues and tumor cells.

(Supplementary Figure 3B)The expression level of KCNQ4 in tumor tissues and tumor cells.


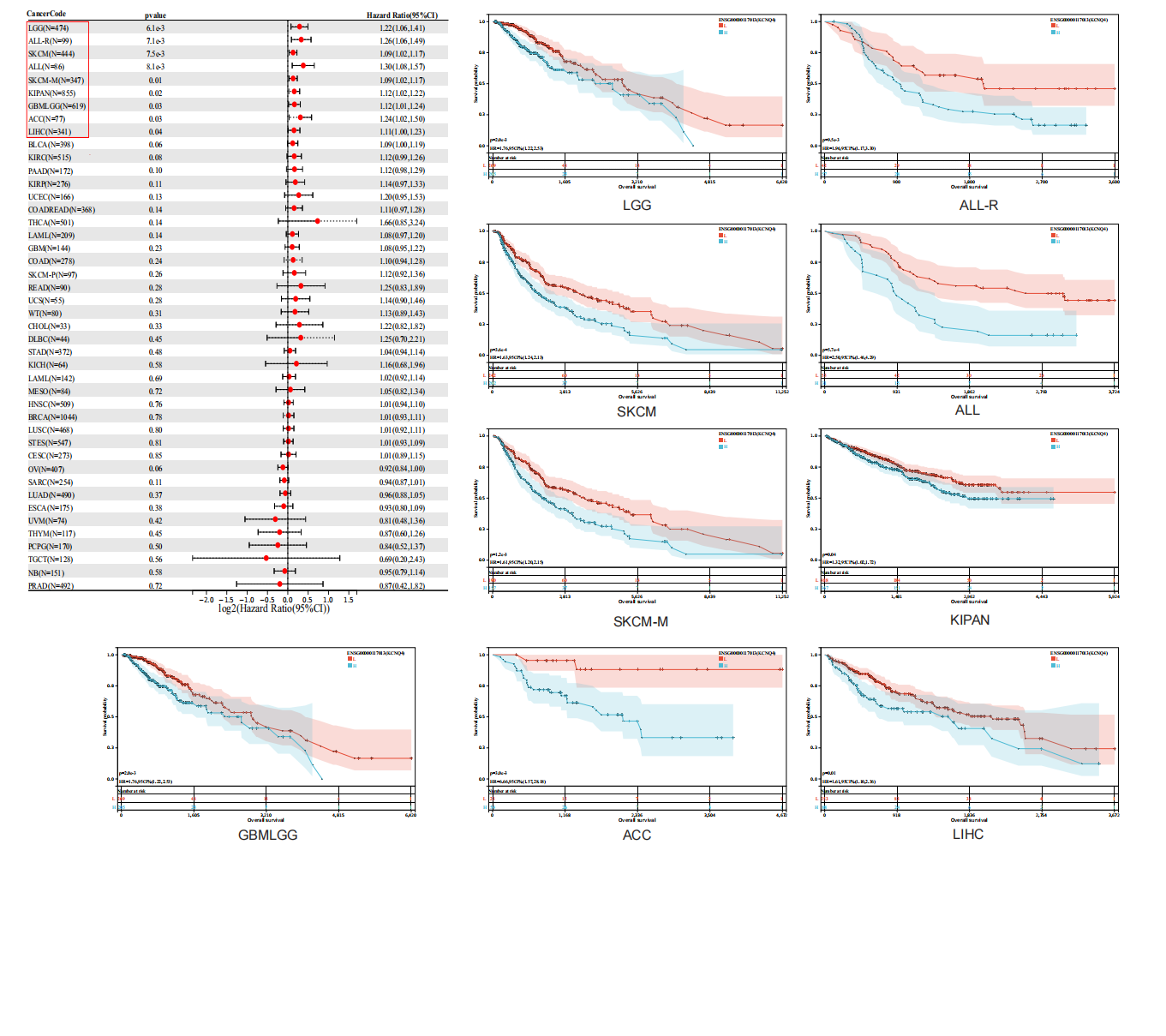
**Supplementary Figure 4 |OS of prognostic analysis of KCNQ4 from UCSC.**


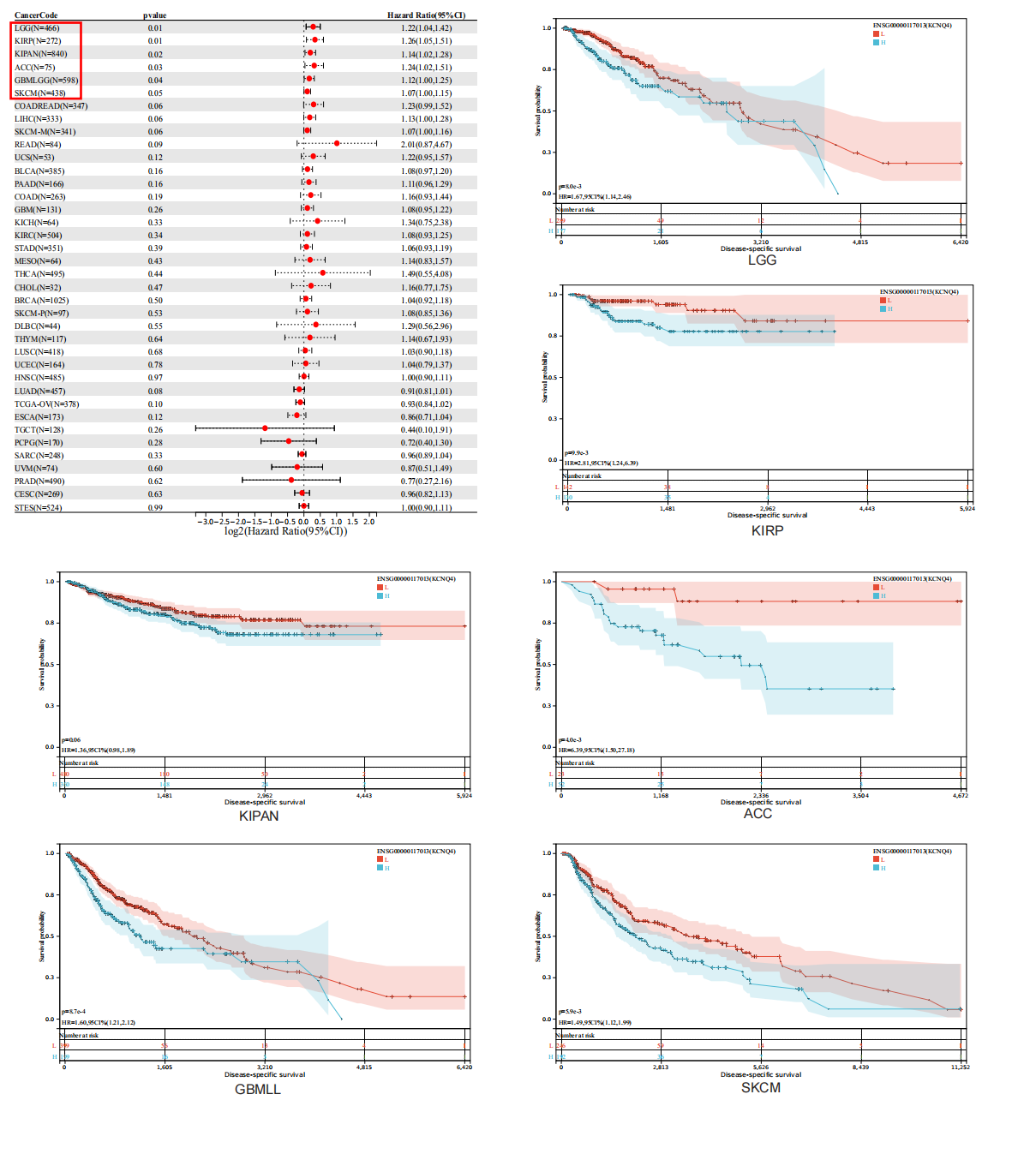
**Supplementary Figure 5 |DSS of prognostic analysis of KCNQ4 from UCSC.**


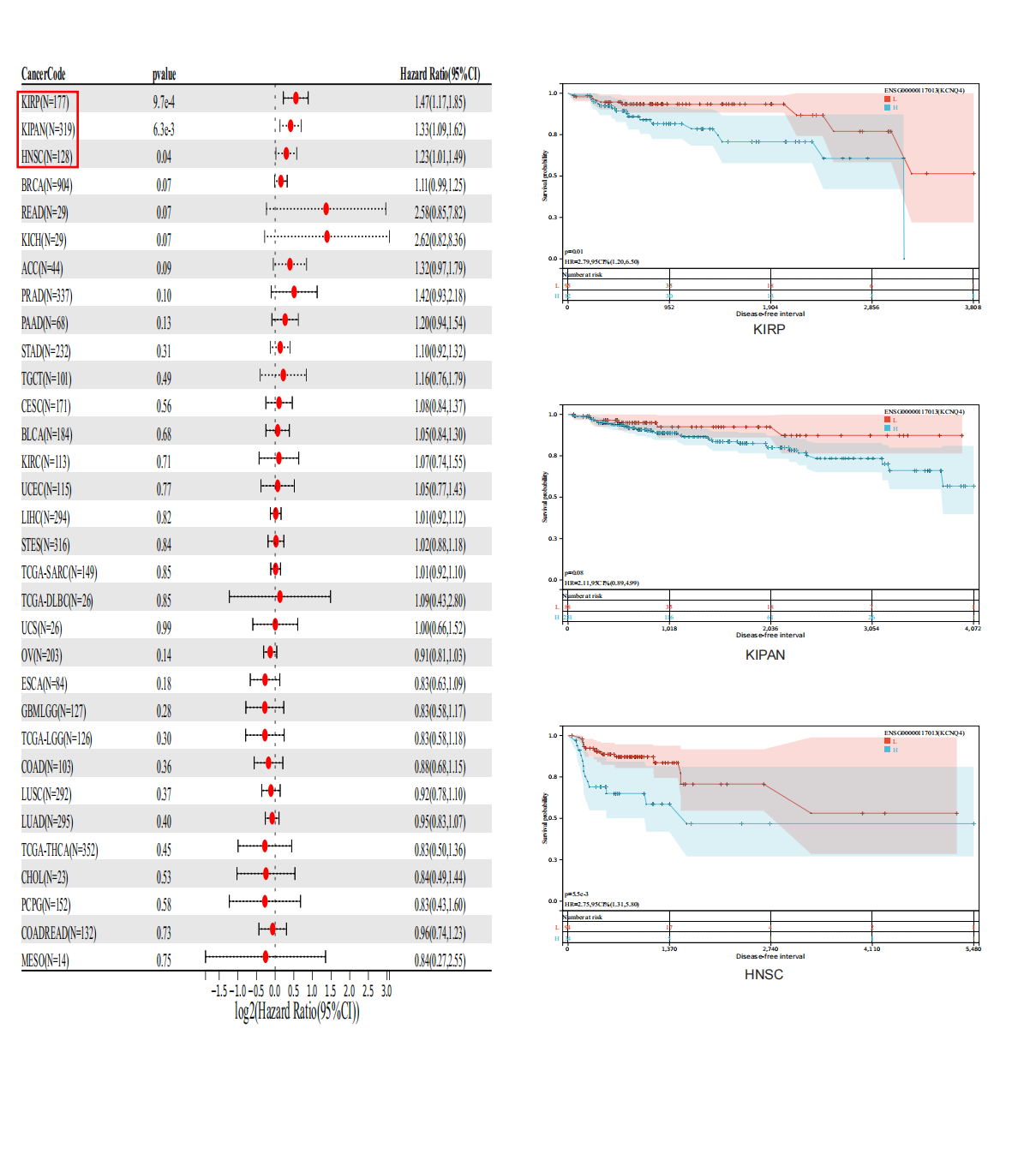
**Supplementary Figure 6 |DFS of prognostic analysis of KCNQ4 from UCSC.**


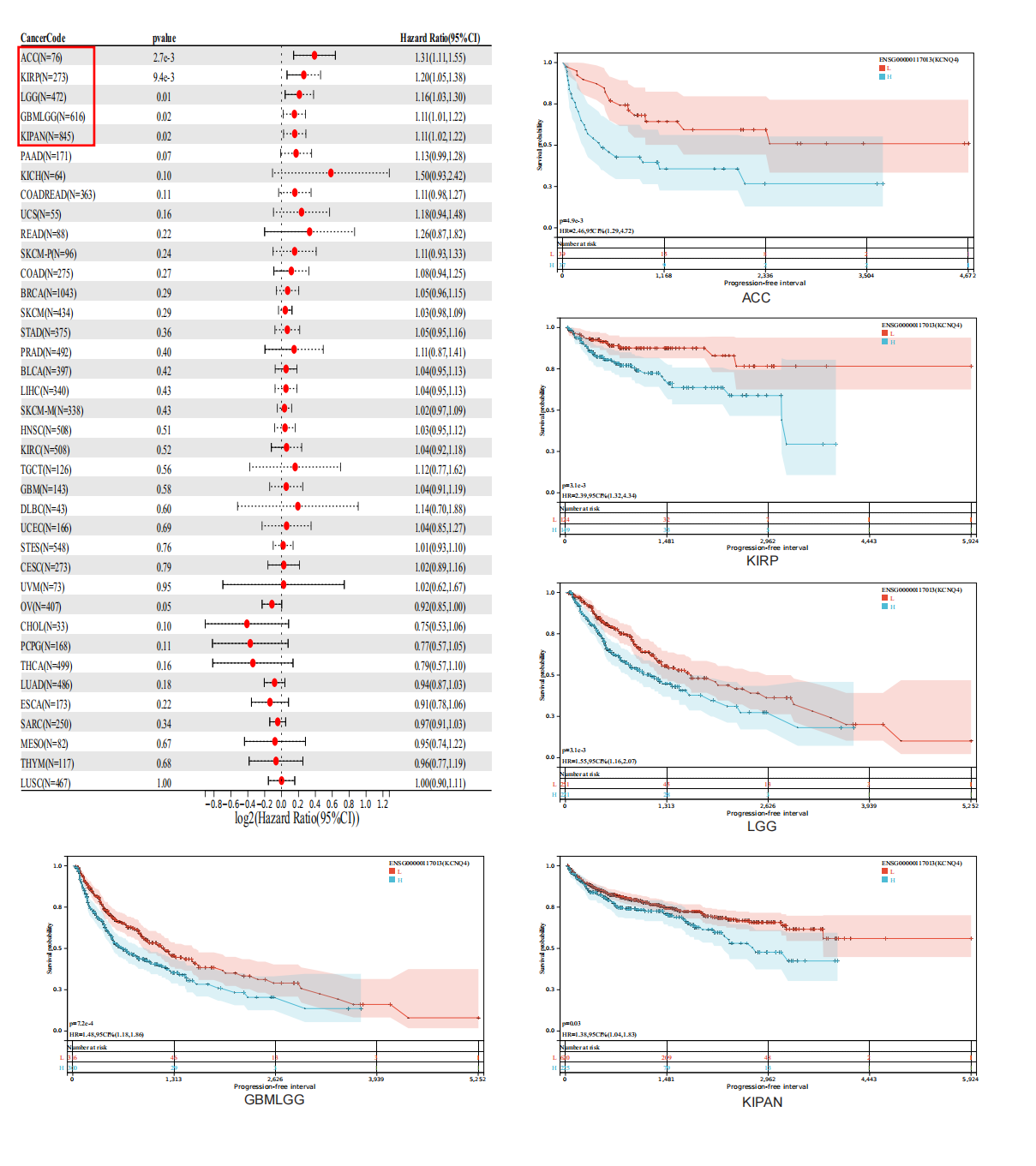


**Supplementary Figure 7 |****PFS of prognostic analysis of KCNQ4 from UCSC.**


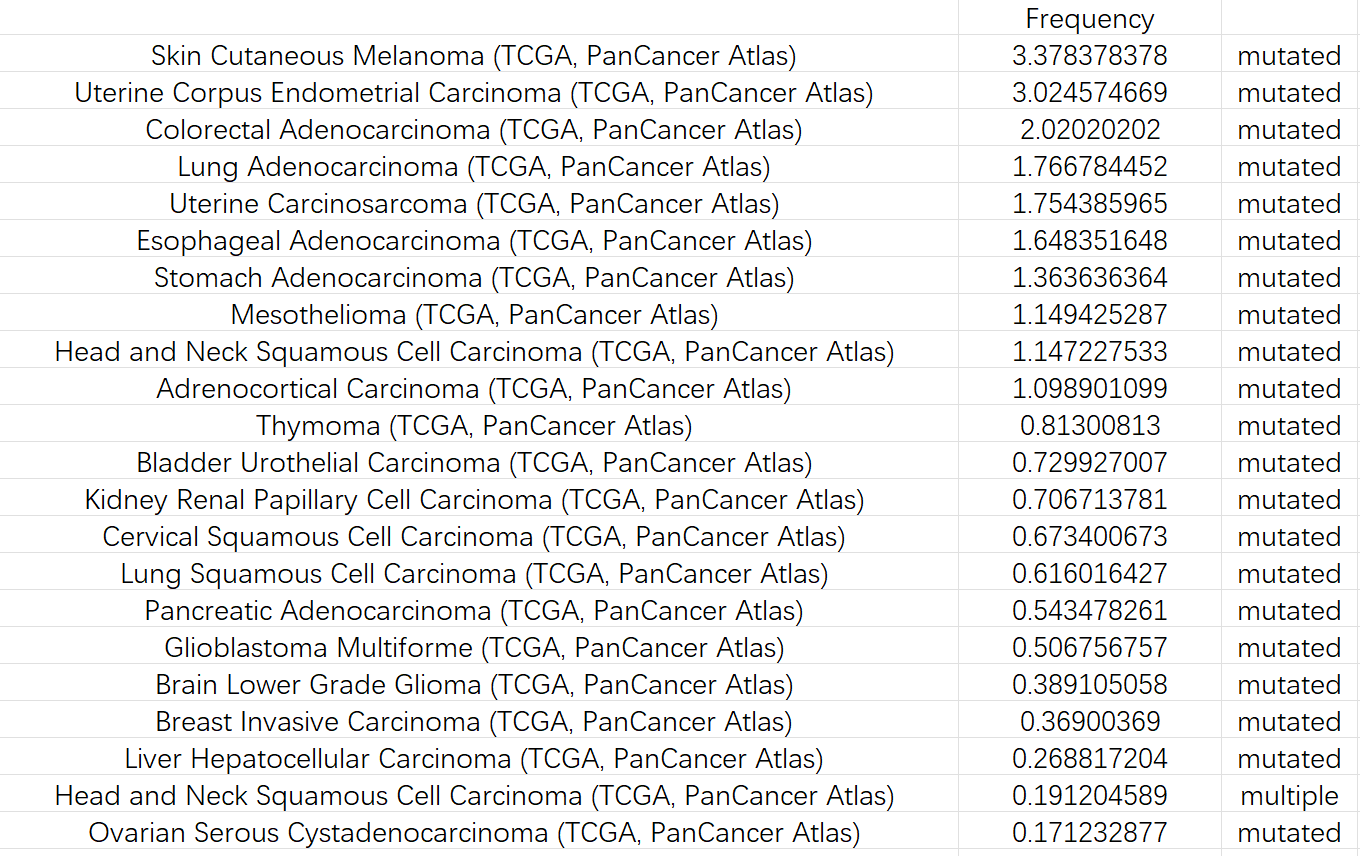
**Supplementary Figure 8|The mutation frequency of KCNQ4 via Bioportal**


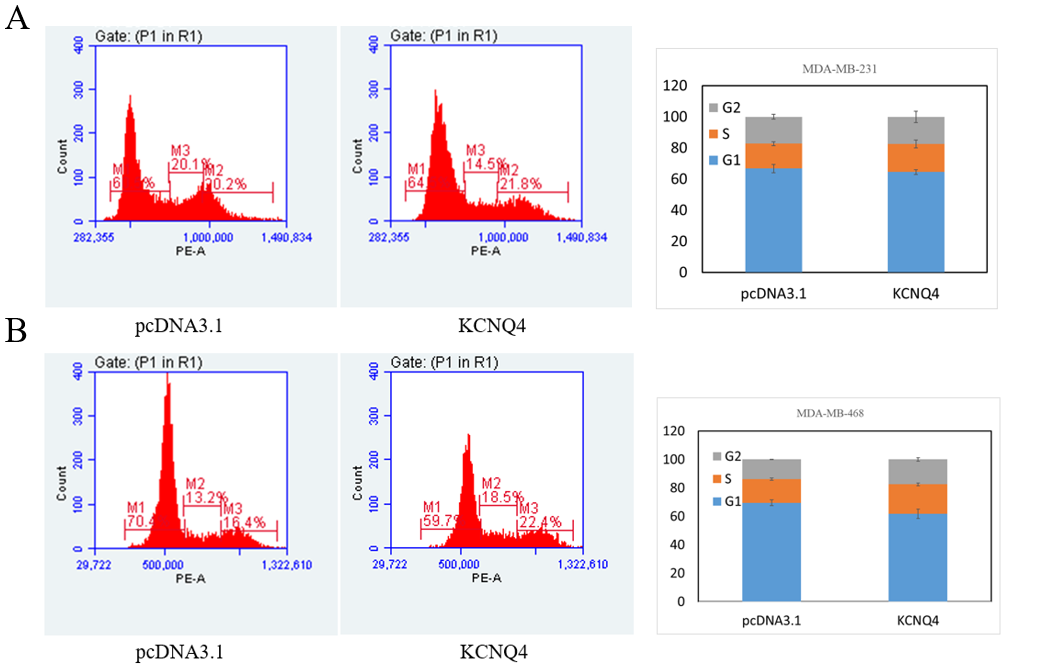


**Supplementary Figure 9|Breast Cell cycle experiment of KCNQ4**

The effect of KCNQ4 on breast cancer cell line MDA-MB-231 was detected by flow cytometry(Supplementary Figure 9A) and the impact of MDA-MB-468 (Supplementary Figure 9B) cycles
